# Cooperative inhibition of RIP1-mediated NF-κB signaling by cytomegalovirus-encoded deubiquitinase and inactive homolog of cellular ribonucleotide reductase large subunit

**DOI:** 10.1371/journal.ppat.1006423

**Published:** 2017-06-01

**Authors:** Ki Mun Kwon, Se Eun Oh, Young Eui Kim, Tae-Hee Han, Jin-Hyun Ahn

**Affiliations:** Department of Molecular Cell Biology, Sungkyunkwan University School of Medicine, Suwon, Republic of Korea; Heinrich Pette Institute, Leibniz Institute for Experimental Virology, GERMANY

## Abstract

Several viruses have been found to encode a deubiquitinating protease (DUB). These viral DUBs are proposed to play a role in regulating innate immune or inflammatory signaling. In human cytomegalovirus (HCMV), the largest tegument protein encoded by UL48 contains DUB activity, but its cellular targets are not known. Here, we show that UL48 and UL45, an HCMV-encoded inactive homolog of cellular ribonucleotide reductase (RNR) large subunit (R1), target receptor-interacting protein kinase 1 (RIP1) to inhibit NF-κB signaling. Transfection assays showed that UL48 and UL45, which binds to UL48, interact with RIP1 and that UL48 DUB activity and UL45 cooperatively suppress RIP1-mediated NF-κB activation. The growth of UL45-null mutant virus was slightly impaired with showing reduced accumulation of viral late proteins. Analysis of a recombinant virus expressing HA-UL45 showed that UL45 interacts with both UL48 and RIP1 during virus infection. Infection with the mutant viruses also revealed that UL48 DUB activity and UL45 inhibit TNFα-induced NF-κB activation at late times of infection. UL48 cleaved both K48- and K63-linked polyubiquitin chains of RIP1. Although UL45 alone did not affect RIP1 ubiquitination, it could enhance the UL48 activity to cleave RIP1 polyubiquitin chains. Consistently, UL45-null virus infection showed higher ubiquitination level of endogenous RIP1 than HA-UL45 virus infection at late times. Moreover, UL45 promoted the UL48-RIP1 interaction and re-localization of RIP1 to the UL48-containing virion assembly complex. The mouse cytomegalovirus (MCMV)-encoded DUB, M48, interacted with mouse RIP1 and M45, an MCMV homolog of UL45. Collectively, our data demonstrate that cytomegalovirus-encoded DUB and inactive R1 homolog target RIP1 and cooperatively inhibit RIP1-mediated NF-κB signaling at the late stages of HCMV infection.

## Author summary

Activation of NF-κB signaling leads to expression of proinflammatory cytokines and chemokines and plays a key role in regulating innate immune response and inflammation to virus infection. HCMV upregulates and downregulates NF-κB signaling during the course of infection. Upregulation of NF-κB signaling may promote viral gene expression or viral dissemination, but its downregulation may be necessary for suppression of excessive immune responses. Recently, it was demonstrated that viral late functions downregulate TNFα- and IL-1β-induced NF-κB activation. However, the viral proteins involved and the underlying mechanisms are not understood. In the present study, we demonstrate that two HCMV proteins, the largest tegument protein harboring deubiquitinase activity and the inactive homolog of cellular ribonucleotide reductase large subunit, cooperatively inhibit RIP1-mediated NF-κB signaling at the late stages of infection. This study for the first time identified RIP1 as a substrate of viral deubiquitinase and highlights the importance of the negative regulation of NF-κB during virus infection.

## Introduction

Human cytomegalovirus (HCMV), which belongs to the β-herpesvirus subfamily, typically causes asymptomatic infections in immunocompetent individuals. HCMV is ubiquitously but latently distributed throughout the world. However, infection of pregnant women often causes congenital infection, and reactivation from latent infection in immunocompromised individuals can lead to life-threatening complications [1]. HCMV is a large, enveloped, double-stranded DNA virus and its 235 kb genome encodes for at least 165 proteins [2]. A structural feature unique to herpesviruses is the presence of a protein layer, called the tegument, between the capsid and the envelope. Upon initial fusion of the viral envelope with the host cell plasma membrane, many of these tegument proteins are delivered into the cytoplasm and the nucleus, where they perform diverse functions including activation of viral gene transcription and antagonization of host intrinsic and innate immunity. Viral tegument proteins are also thought to be involved in capsid transport and virion egress [1, 3, 4].

The herpesvirus deubiquitinase (DUB) was first discovered as an N-terminal fragment of the 336 kDa UL36 tegument protein (also known as VP1/2) of herpes simplex virus-1 (HSV-1) [5]. This DUB domain is conserved in the UL36 equivalents of other herpesviruses [6]. Interestingly, the herpesvirus DUBs bear no structural homology to known eukaryotic DUBs, although the key amino acid residues in the active site are highly conserved [7]. The herpesvirus DUBs appear to play a key role in regulating innate immune and inflammatory signaling. HSV-1 UL36 and Kaposi’s sarcoma-associated herpesvirus (KSHV) ORF64 interact with and deubiquitinate TRAF3 and RIG-I, respectively, inhibiting IRF3 activation [8, 9]. Epstein-Barr virus (EBV) BPLF1 targets TRAF6 and inhibits its ubiquitination, downregulating NF-κB activation during lytic infection [10]. BPLF1 is also known to deubiquitinate PCNA and inhibit trans-lesion synthesis at DNA damage sites [11].

In HCMV, the UL48-encoded tegument protein is a homolog of HSV-1 UL36 and possesses DUB activity. The UL48 DUB was identified using a suicide substrate probe specific for ubiquitin-binding cysteine proteases in virus-infected cells [12]. This DUB, mapped to the first ~350 amino acids of the N-terminal region of pUL48, contains both ubiquitin-specific carboxyl-terminal hydrolase activity and isopeptidase activity that cleaves ubiquitin K11, K48, and K64 linkages [13, 14]. Mutations in active site residues (C24 and H162) completely abolish DUB activity, and the virus containing the UL48 (C24S) gene shows moderately reduced growth in culture cells, demonstrating that the DUB activity of HCMV can influence viral replication [14]. Like the UL36 DUBs of HSV-1 and Pseudorabies virus (PRV) [15, 16], the UL48 DUB has autocatalytic activity that regulates its own stability [17].The N-terminal DUB-containing region from UL48 is required for virion stability and efficient virus entry, although the associated DUB activity appears not to be critical [17]. Despite accumulating evidence that DUB activity and the DUB-containing region of UL48 are required for efficient viral growth, whether the UL48 DUB, like the equivalents of α and γ-herpesviruses, plays a role in regulation of innate immune or inflammatory signaling is not known yet. UL48 contains the nuclear localization signal (NLS) that is essential for viral growth [17, 18], suggesting a critical role of UL48 in the nucleus or in routing of the capsid to the nuclear pore as seen in infection with HSV-1 UL36 mutant virus [19, 20].

The nuclear factor-kappa B (NF-κB) transcription factors are critical regulators of host cell’s early responses to viral infection [21]. HCMV infection can induce NF-κB’s transcriptional activity, which subsequently drives expression of a number of different proinflammatory cytokines and chemokines. There is increasing evidence that HCMV can inhibit NF-κB signaling during lytic infection. This negative regulation may be necessary to suppress excessive immune responses that are detrimental to viral infection. Although viral downregulation of NF-κB signaling in the early stages of infection has been confirmed, it is believed that late virus functions are critical to suppress NF-κB signaling. UL26 is an early viral protein that initially localizes to the nucleus, but becomes cytoplasmic as the infection progresses and eventually localizes to virion assembly sites [22, 23]. UL26 is known to antagonize NF-κB activation induced by TNFα by attenuating IKK phosphorylation and subsequent IκBα degradation [24].

The HCMV UL45 gene encodes an inactive homolog of cellular ribonucleotide reductase (RNR) large subunit (R1), which is contained in the tegument. The function of UL45 still remains largely unknown. Analysis of the VR1814-derived bacmid (FIX-BAC) reconstituted recombinant virus showed that the UL45-null mutant virus normally grows in endothelial cells [25] but is defective in viral particle accumulation at low multiplicities of infection (MOI) and in spreading in fibroblasts [26]. The growth defect of UL45-null virus did not appear to result from a reduced dNTP supply [26]. In mouse CMV (MCMV), M45, a homolog of HCMV UL45, interacts with mouse receptor-interacting protein kinase 1 (mRIP1) and inhibits mRIP1-mediated signaling pathways, including activation of NF-κB after stimulation of TNFR1 and TLR3 [27]. M45 also blocks mRIP1-independent NF-κB activation and cytokine production after stimulation of IL-1R and TLRs and binds to and induces lysosomal degradation of NEMO by targeting it to autophagosomes [28]. Furthermore, M45 is also known to inhibit cytosolic DNA sensor DAI-mediated NF-κB activation through RIP homotypic interaction motif (RHIM)-dependent interaction [29]. Meanwhile, M45 acts as a suppressor of virus-induced or TNFα-induced cell death [30, 31]. M45 inhibits a caspase-independent form of programmed necrosis (necroptosis), which depends on the adaptor kinase RIP3 and DAI [32, 33]. It is not known whether UL45 has activity similar to M45 in regulating the host’s immune responses.

In this study, we identified RIP1 as a cellular target of HCMV-encoded deubiquitinase UL48. We provide evidence that RIP1 is also targeted by UL45, an HCMV-encoded inactive homolog of cellular RNR R1, and that UL48 and UL45 cooperatively suppress RIP1-mediated NF-κB signaling at the late stages of viral infection.

## Results

### HCMV UL48 interacts with RIP1

**Fig 1:**
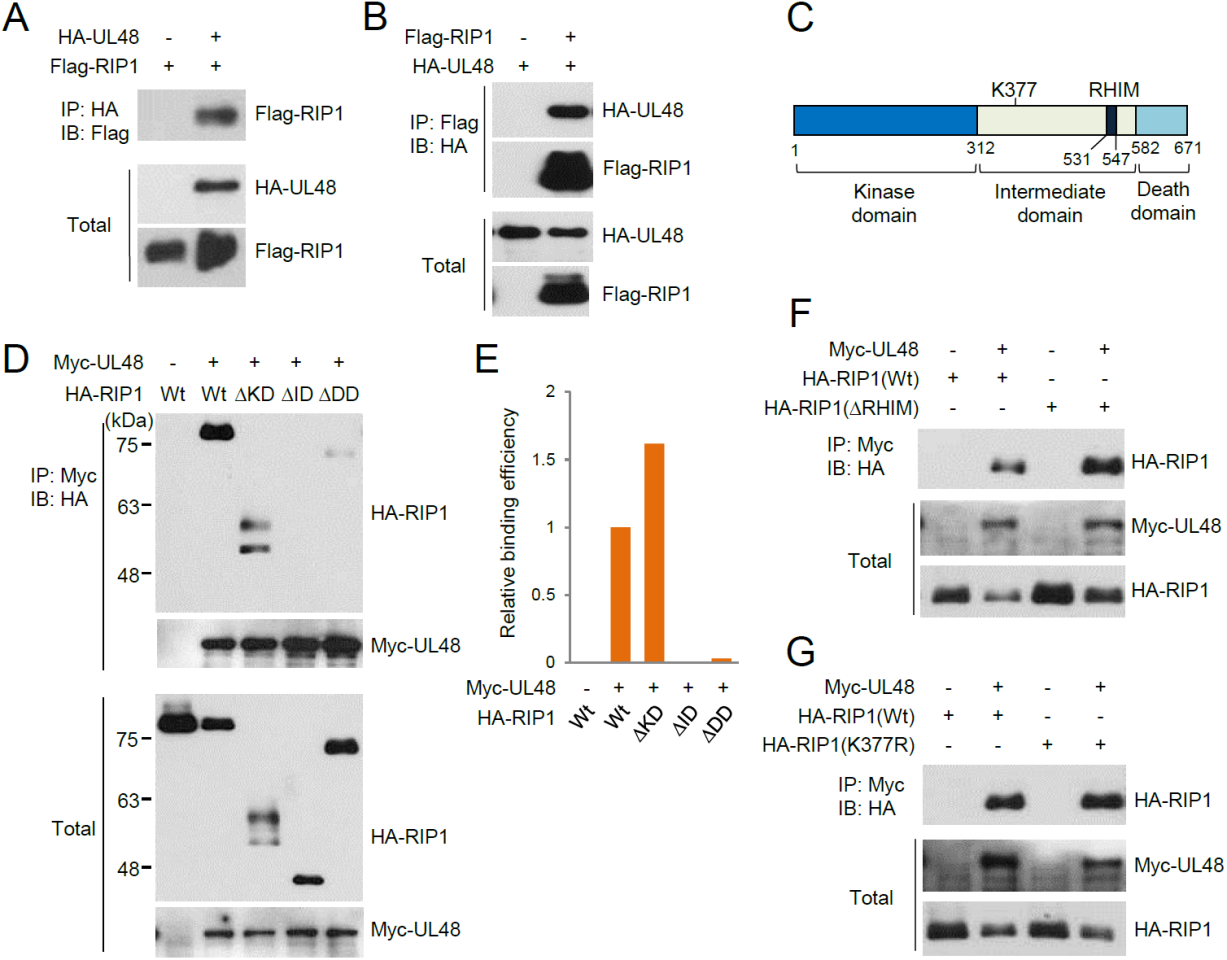
Interaction of UL48 with RIP1 in CoIP assays. (A and B) 293T cells were co-transfected with plasmid expressing HA-UL48 and Flag-RIP1 as indicated. At 24 h after transfection, total cell lysates were immunoprecipitated with anti-HA (A) or anti-Flag (B) antibody, followed by immunoblotting with anti-flag (A) or anti-HA (B) antibody. The protein levels of HA-UL48 and Flag-RIP1 proteins in total cell lysates were also determined by immunoblotting. (C) The domain structure of RIP1 consists of the N-terminal kinase domain (KD), the intermediate domain (ID), and the C-terminal death domain (DD). The TNFα-induced K63-linked polyubiquitination site (K377) and the RHIM within the internal domain are indicated. (D to G) 293T cells were co-transfected with plasmids expressing Myc-UL48 and HA-RIP1 (wild-type or mutants). At 24 h after transfection, total cell lysates were immunoprecipitated with anti-Myc antibody, followed by immunoblotting with anti-HA antibody. The levels of Myc-UL48 and HA-RIP1 proteins in total cell lysates were also determined by immunoblotting. The amounts of wild-type and mutant HA-RIP1 proteins co-immunoprecipitated over the input amounts of proteins in (D) were quantitated by counting using ImageJ (NIH) and the relative binding efficiency is shown as a graph in (E). RIP1 ΔKD appeared as a doublet and the ratio was dependent on the conditions of cell lysate preparation.

Since RIP1 is a critical mediator of NF-κB signaling and K63-linked polyubiquitination of RIP1 is an important process involved in this signaling, we tested whether the HCMV UL48 deubiquitinase targets RIP1. When CoIP assays were performed in co-transfected 293T cells, UL48 was found to interact with RIP1 (Fig 1A and 1B). RIP1 has three domains: the N-terminal kinase domain (KD), the central intermediate domain (ID), and the C-terminal death domain (DD) (Fig 1C). Three RIP1 deletion constructs (ΔKD, ΔID, and ΔDD) were employed for similar CoIP assays to determine the UL48-binding region of RIP1. The results revealed that both the intermediate and death domains of RIP1 are required for efficient RIP1 binding (Fig 1D and 1E). The intermediate domain contains the TNFα-induced Lys63 (K63)-linked polyubiquitination site (K377) [34] and the RHIM. To further investigate the effect of the RHIM and ubiquitination of RIP1 on the pUL48-RIP1 interaction, RHIM-deleted (ΔRHIM) and K377R mutants were also used in CoIP assays. The results showed that pUL48 still interacts with these ΔRHIM and K377R mutant RIP1 proteins (Fig 1F and 1G). Taken together, these results demonstrate that UL48 interacts with RIP1 through the intermediate and death domains, but this interaction does not require presence of the RHIM and polyubiquitination at K377 of RIP1.

### UL48 inhibits RIP1-mediated NF-κB activation

**Fig 2:**
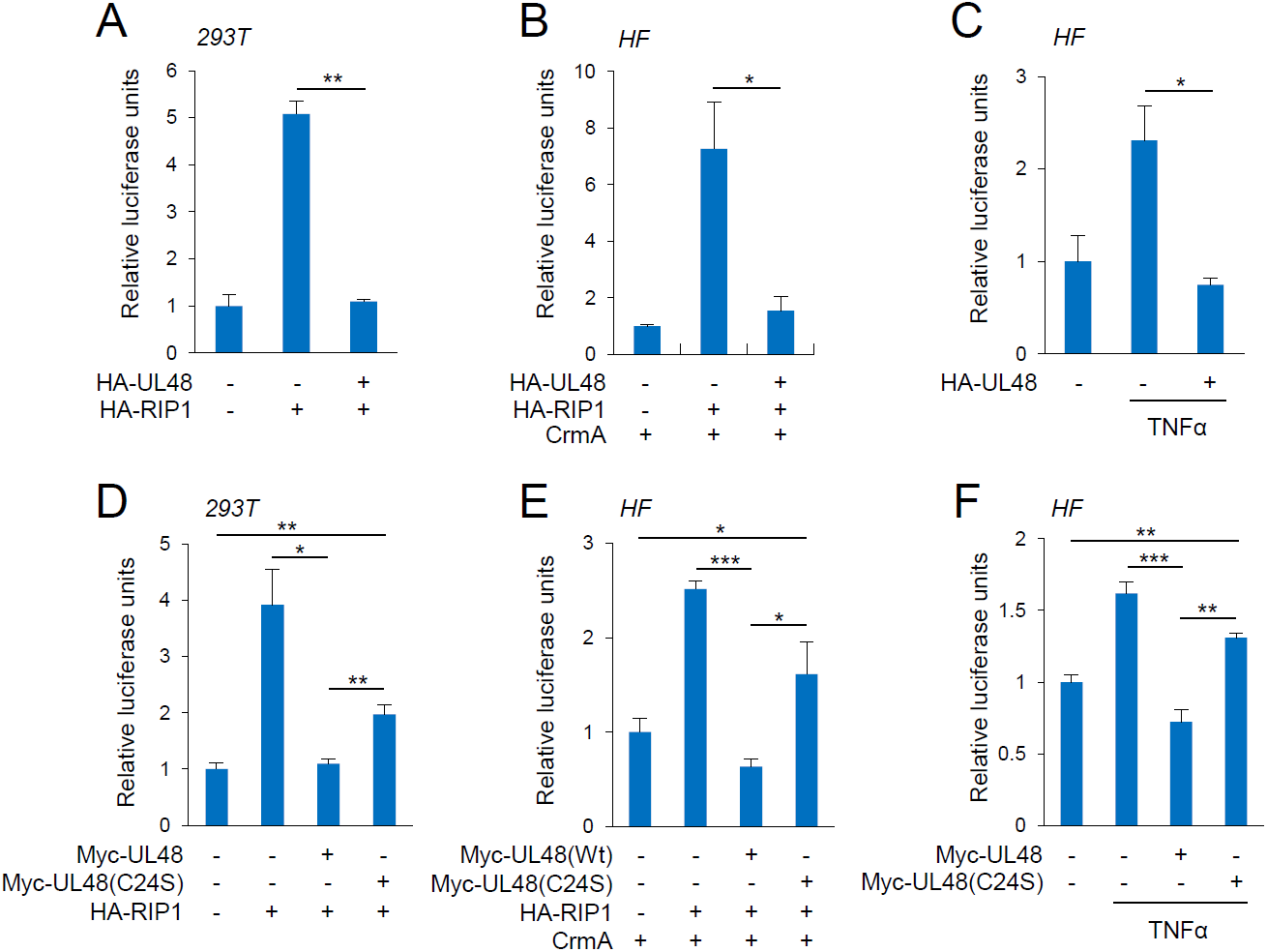
Inhibition of RIP1-mediated NF-κB activation by UL48 in reporter assays. (A) 293T cells in six-well plates were co-transfected using PEI with 0.1 μg of NF-κB-luciferase reporter plasmid, 0.5 μg of SV2-β-galactosidase reporter plasmid in which β-galactosidase expression is driven by the SV40 promoter and enhancer (for normalization), and 0.5 μg plasmids for HA-UL48 or HA-RIP1 as indicated. In all co-transfection assays, empty vectors were used to make the total amount of DNA transfected equal. At 24 h after transfection, total cell lysates were prepared and assayed for luciferase activity. (B and C) HF cells in six-well plates were co-transfected using OmicsFect with 0.5 μg of NF-κB-luciferase reporter plasmid, 0.5 μg of SV2-β-galactosidase reporter plasmid, and 1 μg plasmid for HA-UL48 or 0.5 μg HA-RIP1. Plasmid (0.5 μg) for CrmA (caspase inhibitor) was added to avoid apoptotic cell death due to RIP1 overexpression in (B) and TNFα (50 ng/ml) was added for 8 h before cell harvest in (C). (D) Luciferase reporter assays were performed in 293T cells as in (A) including the C24S mutant of Myc-UL48. (E and F) HF cells were co-transfected using electroporation with 0.5 μg of NF-κB-luciferase and 0.5 μg of SV2-β-galactosidase reporter plasmids and 0.5 μg of plasmids for Myc-UL48 (wild-type or C24S mutant), HA-RIP1, and CrmA as indicated. Total cell lysates were prepared 24 h after transfection and assayed for luciferase activity. TNFα (50 ng/ml) was treated for 8 h before cell harvest in (F). Luciferase activity was normalized with β-galactosidase activity. Shown are mean values with the standard errors of luciferase activity of three independent assays.

Luciferase reporter assays were performed to investigate the effect of UL48 on RIP1-mediated NF-κB activation. UL48 expression effectively inhibited RIP1-mediated NF-κB activation in 293T cells (Fig 2A). HCMV permissive HF cells were more sensitive to apoptotic cell death induced by RIP1 overexpression than other cell types. Therefore, similar reporter assays were performed in HF cells with co-transfection of CrmA, an inhibitor of caspases 1 and 8. The results showed that the NF-κB activity induced by RIP1 expression was effectively inhibited by UL48 in HF cells (Fig 2B). TNFα treatment of HF cells moderately increased NF-κB activity, but this elevation was also inhibited by UL48 (Fig 2C). We next investigated whether the DUB catalytic activity of UL48 is required for this suppression using the C24S mutant in which the Cys24 active site was replaced with Ser [14]. The results of reporter assays showed that the inhibitory effect of UL48(C24S) on RIP1-mediated and TNFα-induced NF-κB activation was reduced to 30% to 60% that of wild-type UL48 (Fig 2D to 2F). The expression levels of wild-type and C24S mutant UL48 proteins were comparable and the RIP1 modification (probably ubiquitination) was reduced by wild-type UL48 but not by C24S mutant in transfected cells (S1A Fig). These results suggest that the DUB activity of UL48 is largely required for inhibition of RIP1-mediated NF-κB activation.

### Interaction of HCMV UL45 with RIP1

**Fig 3:**
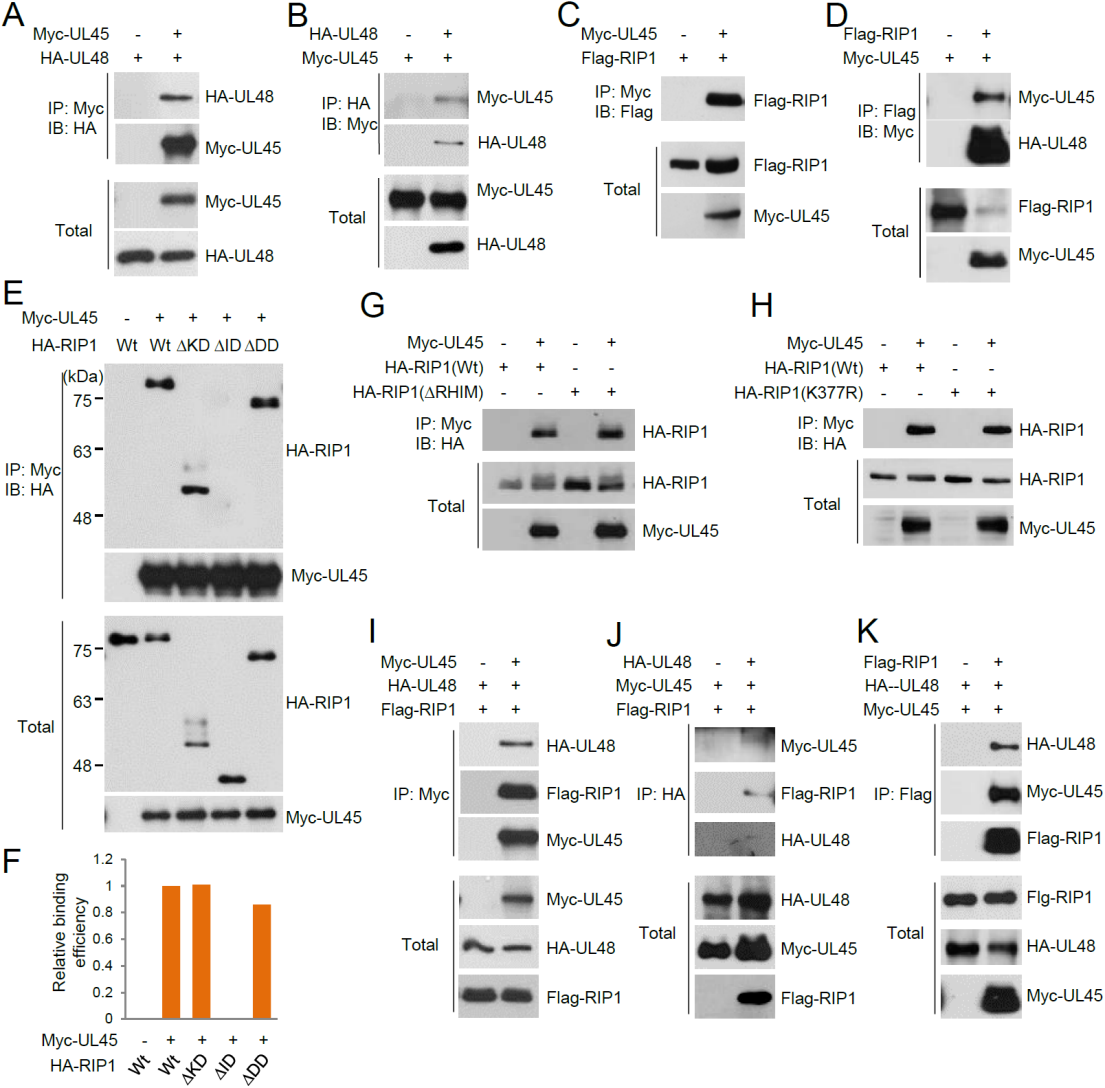
Interaction of UL45 with RIP1 in CoIP assays. (A to D) 293T cells were co-transfected with plasmids expressing Myc-UL45, HA-UL48, or Flag-RIP1 as indicated. At 24 h after transfection, total cell lysates were immunoprecipitated with anti-Myc (A and C), anti-HA (B), or anti-Flag (D) antibody and immunoblotting assays were performed with anti-HA (A), anti-Myc (B and D), or anti-Flag (C) antibody. The protein levels of Myc-UL45, HA-UL48, and Flag-RIP1 proteins in total cell lysates were also determined by immunoblotting. (E to H) 293T cells were co-transfected with plasmids expressing Myc-UL45 and HA-RIP1 (wild-type or mutants). At 24 h after transfection, CoIP assays were performed as in (A). The protein levels of Myc-UL45 and HA-RIP1 proteins in total cell lysates were also determined by immunoblotting. The amounts of wild-type and mutant HA-RIP1 proteins co-immunoprecipitated over the input amounts of proteins in (E) were quantitated using ImageJ (NIH) and the relative binding efficiency is shown as a graph in (F). (I to K) 293T cells were co-transfected with Myc-UL45, HA-UL48, and Flag-RIP1 plasmids as indicated. At 24 h after transfection, total cell lysates were immunoprecipitated with anti-Myc (I), anti-HA (J), or anti-Flag (K) antibody, followed by immunoblotting with anti-HA, anti-Flag, or anti-Myc antibody, as indicated. The levels of Myc-UL45, HA-UL48, and Flag-RIP1 proteins in cell lysates are also shown.

UL48 was first shown to interact with UL45, an inactive homolog of cellular RNR R1, in yeast two-hybrid interaction assays [35]. We also observed this interaction in CoIP assays (Fig 3A and 3B) [17]. Given that UL48 interacted with both RIP1 and UL45 and that M45, an MCMV-encoded homolog of UL45, interacted with mRIP1 [27, 31], the potential for interaction of UL45 with RIP1 was also tested. The results of CoIP assays revealed that UL45 interacts with RIP1 (Fig 3C and 3D).

CoIP assays using the mutant RIP1 constructs revealed that UL45 interacts with RIP1 through the intermediate domain (Fig 3E and 3F). The RHIM was reported to mediate the interaction between M45 and mRIP1 [31]. However, the UL45-RIP1 interaction did not seem to require the RHIM because UL45 did not contain an apparent RHIM, and a RHIM-deleted RIP1mutant also effectively bound to UL45 (Fig 3G). Furthermore, the K377R mutant RIP1 still interacted with UL45, demonstrating that the UL45-RIP1 interaction does not require polyubiquitination at K377 of RIP1 (Fig 3H). When UL45, UL48, and RIP1 were co-expressed, both UL48 and RIP1 were co-precipitated when UL45 was immunoprecipitated (Fig 3I). Similarly, UL45 and RIP1 were co-precipitated with UL48 (Fig 3J), and UL48 and UL45 were co-precipitated with RIP1 (Fig 3K). These results suggest the possibility that both UL48 and UL45 simultaneously target RIP1 and that they may form a complex.

### UL48 and UL45 cooperatively inhibit RIP1-mediated NF-κB activation

**Fig 4:**
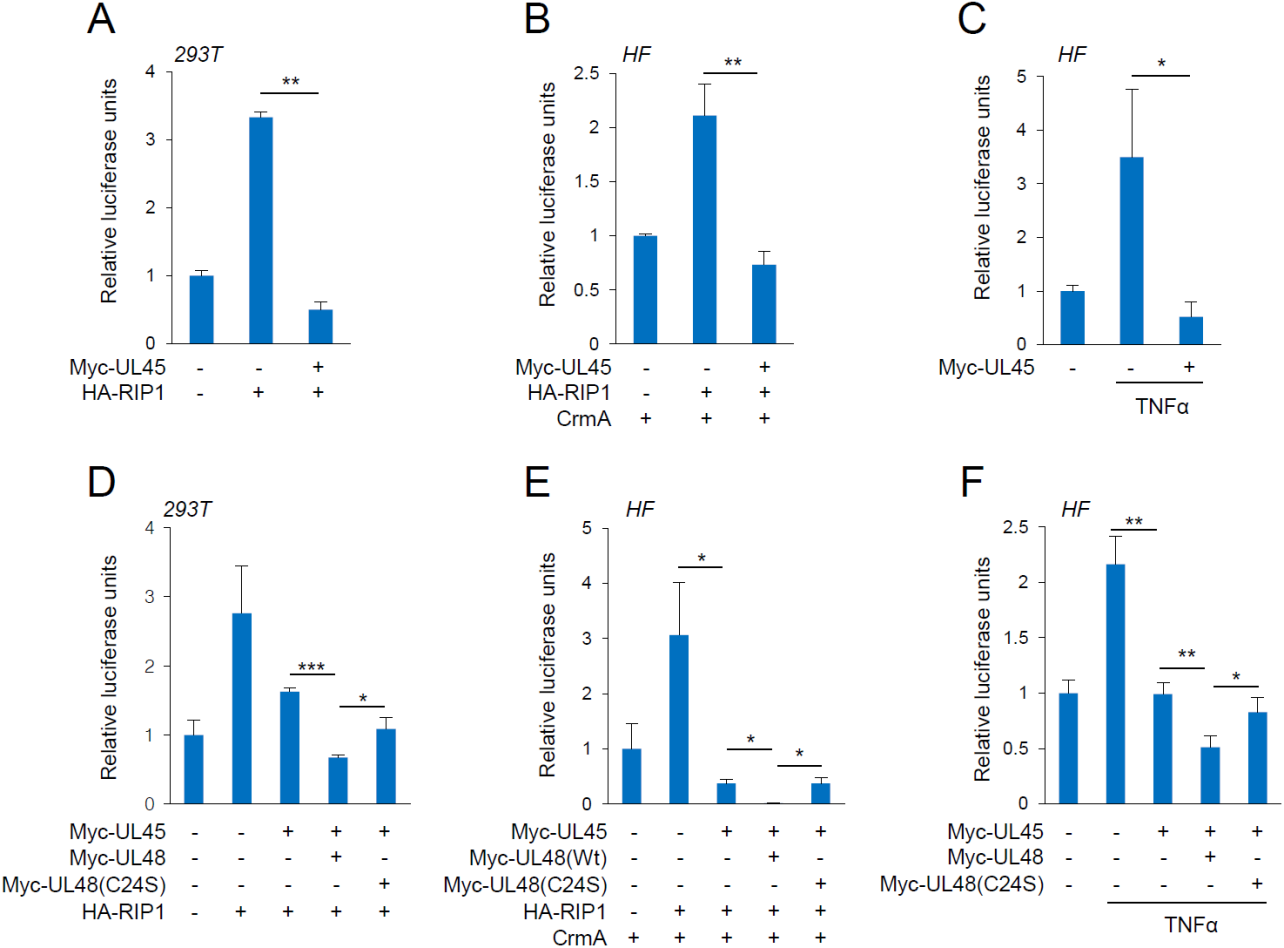
Cooperative inhibition of RIP1-mediated NF-κB activation by UL45 and UL48 in reporter assays. (A) 293T cells in six-well plates were co-transfected using PEI with 0.1 μg of NF-κB-luciferase and 0.5 μg of SV2-β-galactosidase (for normalization) reporter plasmids, and 0.5 μg plasmids for Myc-UL45 and HA-RIP1 as indicated. At 24 h after transfection, total cell lysates were prepared and assayed for luciferase activity. (B and C) HF cells in six-well plates were co-transfected using OmicsFect with 0.5 μg of NF-κB-luciferase and 0.5 μg of SV2-β-galactosidase reporter plasmids and 1 μg plasmid for HA-UL45 or 0.5 μg HA-RIP1. Plasmid (0.5 μg) for CrmA was added in (B) and TNFα (50 ng/ml) was added for 8 h before cell harvest in (C). (D) 293T cells were co-transfected using PEI with NF-κB-luciferase and 0.5 μg of SV2-β-galactosidase reporter plasmids and 0.1 μg of plasmids for Myc-UL45 and Myc-UL48 (wild-type or C24S mutant), or 0.5 μg of HA-RIP1. (E and F) HF cells were co-transfected using electroporation with 0.5 μg of NF-κB-luciferase reporter plasmid, SV2-β-galactosidase reporter plasmid, and plasmids for Myc-UL45, Myc-UL48 (wild-type or C24S mutant), HA-RIP1, and CrmA, as indicated. Total cell lysates were prepared 24 h after transfection and assayed for luciferase activity. TNFα (50 ng/ml) was treated for 8 h before cell harvest in (F). Luciferase activity was normalized with β-galactosidase activity. Shown are mean values with the standard errors of luciferase activity of three independent assays.

We investigated whether, like UL48, UL45 inhibits RIP1-mediated NF-κB activation. In reporter assays performed in 293T and HF cells, NF-κB activity induced by RIP1 expression was effectively inhibited by UL45 (Fig 4A and 4B). UL45 also inhibited NF-κB activation induced by TNFα treatment in HF cells (Fig 4C). We further investigated whether UL48 and UL45 cooperatively affect RIP1-mediated NF-κB activation. In reporter assays, co-expression of UL48 and UL45 more effectively inhibited NF-κB activation by RIP1 than UL45 alone, and this cooperative effect of UL48 and UL45 was largely diminished when the UL48(C24S) mutant was used (Fig 4D and 4E; S1B Fig). This inhibitory effect of UL48 and UL45 was also observed in TNFα-induced NF-κB activation in HF cells (Fig 4F). Collectively, our data demonstrate that UL48 and UL45 may cooperate to inhibit RIP1-mediated NF-κB activation, and that the deubiquitinating activity of UL48 contributes to this repression.

### UL45 contributes to efficient accumulation of viral late proteins at low MOI

**Fig 5:**
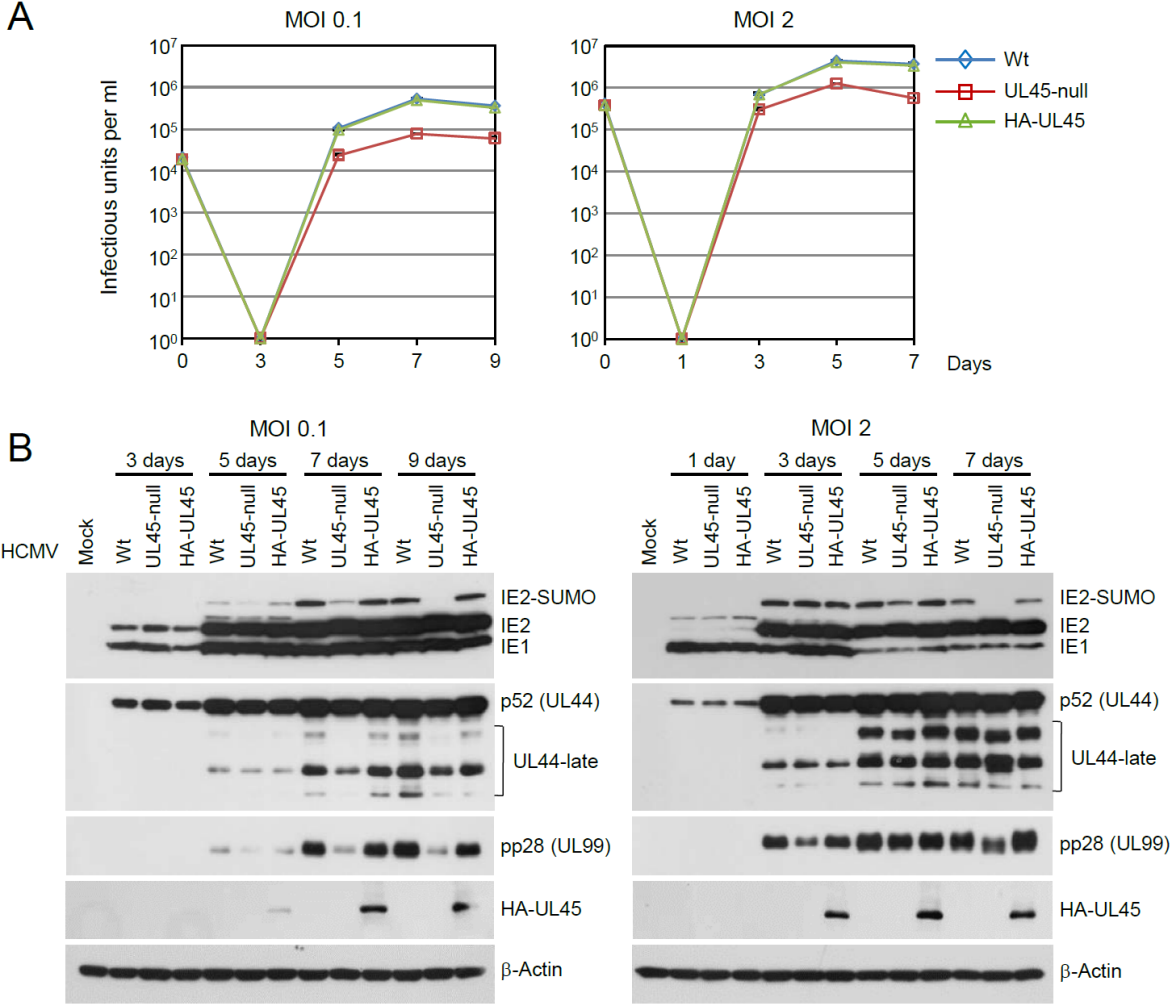
Growth comparison of wild-type, UL45-null, and HA-UL45 Toledo. (A and B) HF cells in 12-well plates were mock infected or infected with Toledo viruses (wild-type, UL45stop mutant, or HA-UL45) at an MOI of 0.1 or 2. Virus titers in the culture supernatants were measured by infectious center assays. The time course results shown represent the amounts of virus produced at the indicated sampling times. The results are the averages of data from two independent assays, and titers at day 0 represent input inocula (A). The total cell lysates from mock-infected or virus-infected cells were prepared at the indicated time points and were subjected to SDS-PAGE and immunoblotting for IE1, IE2, p52 (UL44), pp28 (UL99), HA-UL45, and β-actin (B).

To address the significance of the RIP1 targeting by UL48 and UL45 during HCMV infection, recombinant Toledo viruses that do not express UL45 (UL45-null) or express HA-tagged UL45 (HA-UL45) were produced using bacmid mutagenesis (S2 Fig). The growth curves of wild-type, UL45-null, and HA-UL45 viruses were compared in HF cells at an MOI of 0.1 or 2. The viruses released in the culture supernatants and associated with the cells were collected and pooled at various time points after infection, and titers of the infectious progeny virions were determined by infectious center assays. At an MOI of 0.1, the peak titers of UL45-null virus on day 7 was seven-fold lower than those of wild-type and HA-UL45 viruses (Fig 5A, left), while, at an MOI of 2, UL45-null virus produced about three-fold fewer progeny virions on day 5 than wild-type and HA-UL45 viruses (Fig 5A, right). The results of immunoblotting assays showed that the reduced growth of the UL45-null virus at low MOI correlated with the low level accumulation of viral late proteins, such as the late forms encoded by UL44 and the pp28 (encoded by UL99) true late protein (Fig 5B, left), while this difference was reduced at an MOI of 2 (Fig 5B, right). We also observed the reduced accumulation of the SUMO-modified forms of IE2 in UL45-null virus infection, which was often observed in mutant viruses with a moderate growth defect [14, 36]. Collectively, the analysis of UL45-null and HA-UL45 virus growth patterns suggests that UL45 contributes to viral growth in cultured cells at low MOI by affecting the late stages of the virus life cycle and that HA-UL45 virus has a similar growth pattern as wild-type virus.

### Formation of a complex containing UL48, UL45, and RIP1 during viral infection

**Fig 6:**
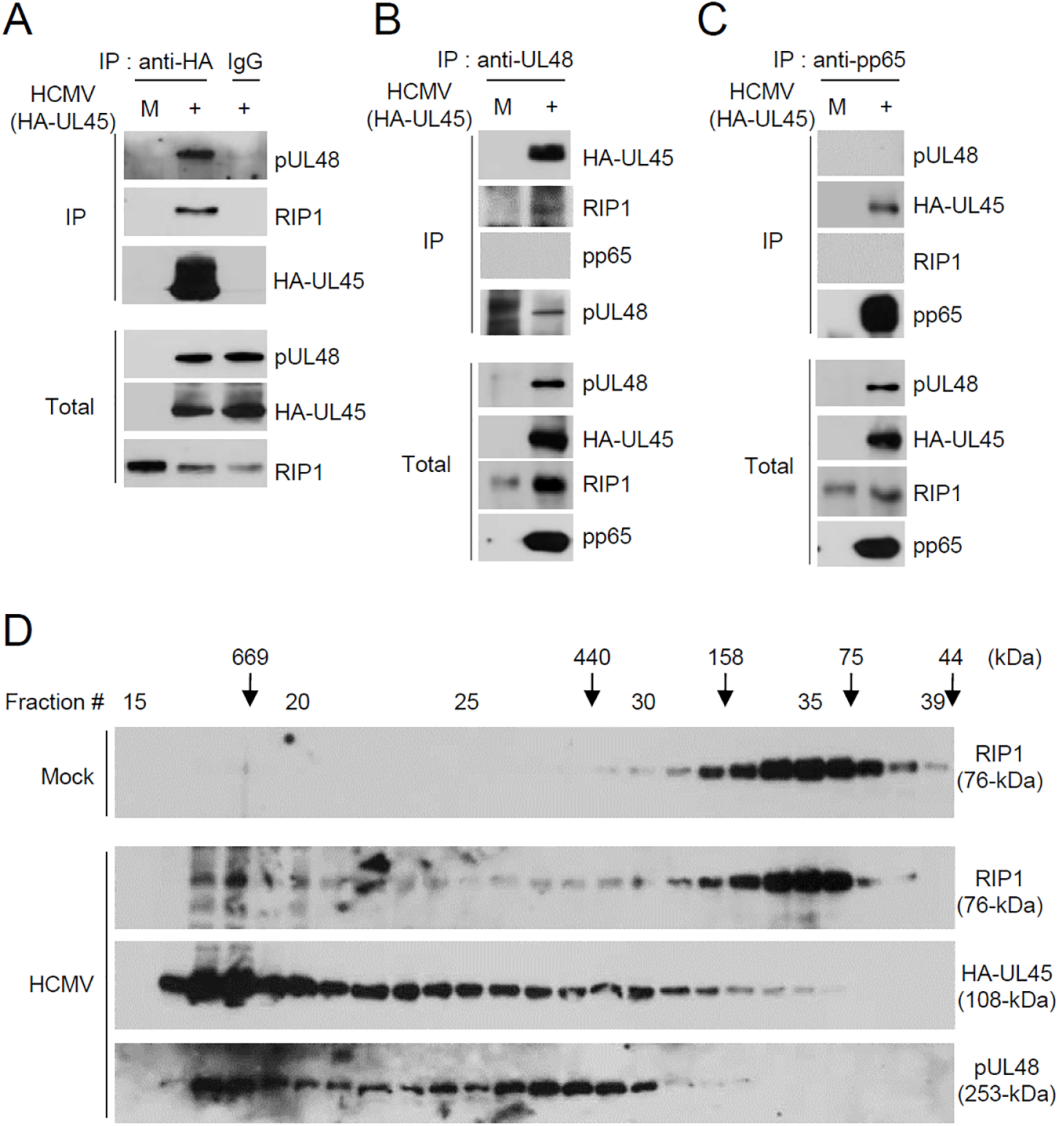
Association of HA-UL45 with UL48 and RIP1 during HA-UL45 virus infection. (A to C) HF cells were mock infected or infected with HA-UL45 Toledo virus for 72 h at an MOI of 1. At 72 h after infection, total cell lysates were immunoprecipitated with anti-HA (A), anti-UL48 (B), or anti-pp65 (UL83) antibody and immunoblotting was performed with indicated antibodies. The protein levels of HA-UL45, pUL48, pp65, and RIP1 in total cell lysates were determined by immunoblotting. (D) Gel filtration chromatography. HF cells were mock infected or infected with HA-UL45 virus for 72 h at an MOI of 3. Total cell lysates were prepared and loaded onto a Superose6 10/300 GL column (GE Healthcare) pre-equilibrated with CoIP buffer. The proteins were eluted at 0.5 ml/min. Fifteen microliters of each fraction was analyzed by immunoblotting with anti-RIP1, anti-HA, or anti-UL48 antibody. Apparent molecular mass was determined after column calibration with standard proteins [thyroglobulin (669 kDa), ferritin (440 kDa), aldolase (158 kDa), conalbumin (75 kDa), and ovalumin (44 kDa)] in the Gel Filtration Calibration Kit (GE Healthcare). The elution positions of these proteins are indicated at the top.

Since each of UL48, UL45, and RIP1 was co-precipitated with the other two proteins in co-transfected cells, the possible formation of a complex containing these three proteins during viral infection was investigated using HA-UL45 virus. When HF cells were infected with HA-UL45 virus, immunoprecipitation of HA-UL45 co-precipitated both UL48 and RIP1 (Fig 6A) and immunoprecipitation of UL48 co-precipitated both HA-UL45 and RIP1 (Fig 6B). In a control CoIP experiment, however, immunoprecipitation of abundant pp65 viral protein did not co-precipitated UL48 and RIP1, although it unexpectedly pull-downed HA-UL45 (Fig 6C). These results suggest that RIP1 may exist in a protein complex containing UL48 and UL45. Furthermore, the result of gel filtration chromatography demonstrated that in HA-UL45 virus-infected cells, the RIP1 fractions shifted to higher molecular mass fractions compared to those in mock-infected cells, and that these high molecular mass fractions (>400 kDa) contained HA-UL45 and UL48 (Fig 6D). These results suggest that both UL48 and UL45 interact with RIP1, and a complex containing UL48, UL45, and RIP1 may be produced during HCMV infection.

**Fig 7:**
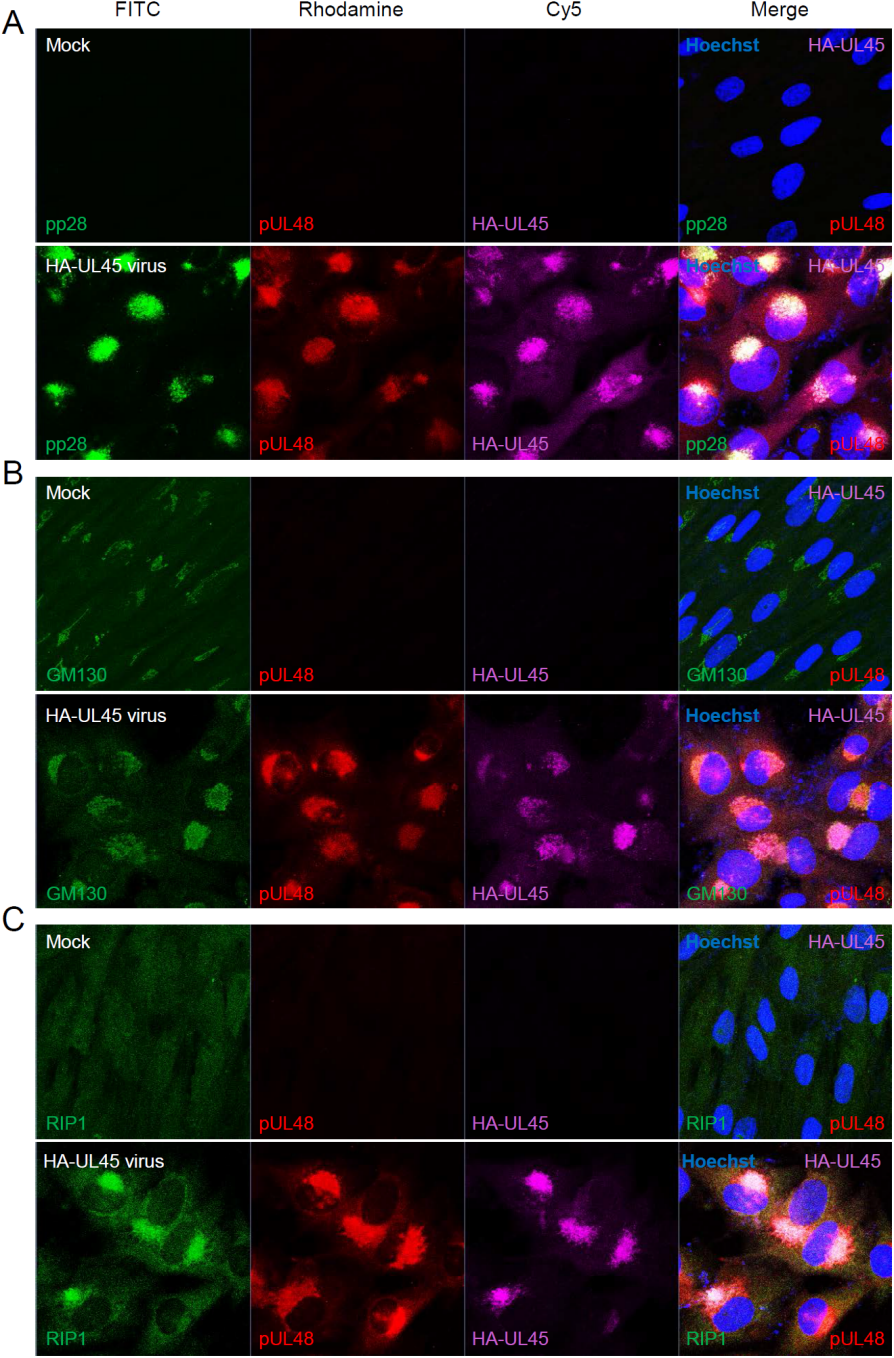
Colocalization of HA-UL45, UL48, and RIP1 in HA-UL45 virus-infected cells. (A to C) HF cells were mock-infected or infected with HA-UL45 Toledo virus for 96 h at an MOI of 1. Cells were fixed with cold methanol and then subjected to triple-label IFA with anti-HA, anti-pp28, and anti-UL48 antibodies (A), with anti-HA, anti-UL48, and anti-GM130 antibodies (B), or with anti-HA, anti-UL48, and anti-RIP1 antibodies (C). FITC-labeled anti-mouse IgG, Rhodamine/Red X-coupled anti-rabbit IgG, and Cy5-conjugated anti-rat IgG antibodies were used for visualization. Hoechst stain was used to stain cell nuclei. The images were obtained by confocal microscopy. Three side-by-side panels of signal-labeled images and a fourth panel with a merged image (including DNA staining) are shown.

We also investigated the localization patterns of UL48, HA-UL45, and RIP1 in cells infected with HA-UL45 virus. Human *γ*-globulin was used as a blocking agent for HCMV Fc receptors, which are recognized by rabbit antibodies in virus-infected cells at the late stages of infection (S3 Fig). When triple-label IFA was performed to visualize UL48, HA-UL45, and pp28 at 96 h after infection, UL48 and HA-UL45 were largely colocalized with pp28 in the cytoplasmic compartments, while a small fraction was also detected in the nucleus as punctate forms (Fig 7A). Since pp28 is localized in the cytoplasmic virion assembly complex (cVAC) [37], the association of UL48 and HA-UL45 with the cVAC was further investigated by co-staining GM130, a Golgi-associated cellular marker for the cVAC [38]. The results demonstrated that UL48 and HA-UL45 are largely colocalized with GM130 in the cVAC (Fig 7B). We next performed similar triple-label IFA to visualize UL48, HA-UL45, and RIP1 in virus-infected cells. In mock-infected cells, endogenous RIP1 was detected at very low levels throughout the cells as a diffuse form. However, in virus-infected cells, RIP1 was slightly stabilized and accumulated in the UL48 and HA-UL45-containing cVAC (Fig 7C). In two-color merge images, RIP1 appeared to be more effectively colocalized with HA-UL45 than UL48 (S4 Fig). These IFA data indicate that RIP1 is colocalized with UL48 and UL45 in the cVAC, supporting that UL48 and UL45 form a complex with RIP1 during viral infection.

### UL48 and UL45 inhibit TNFα-induced NF-κB activation in the late stages of infection

**Fig 8:**
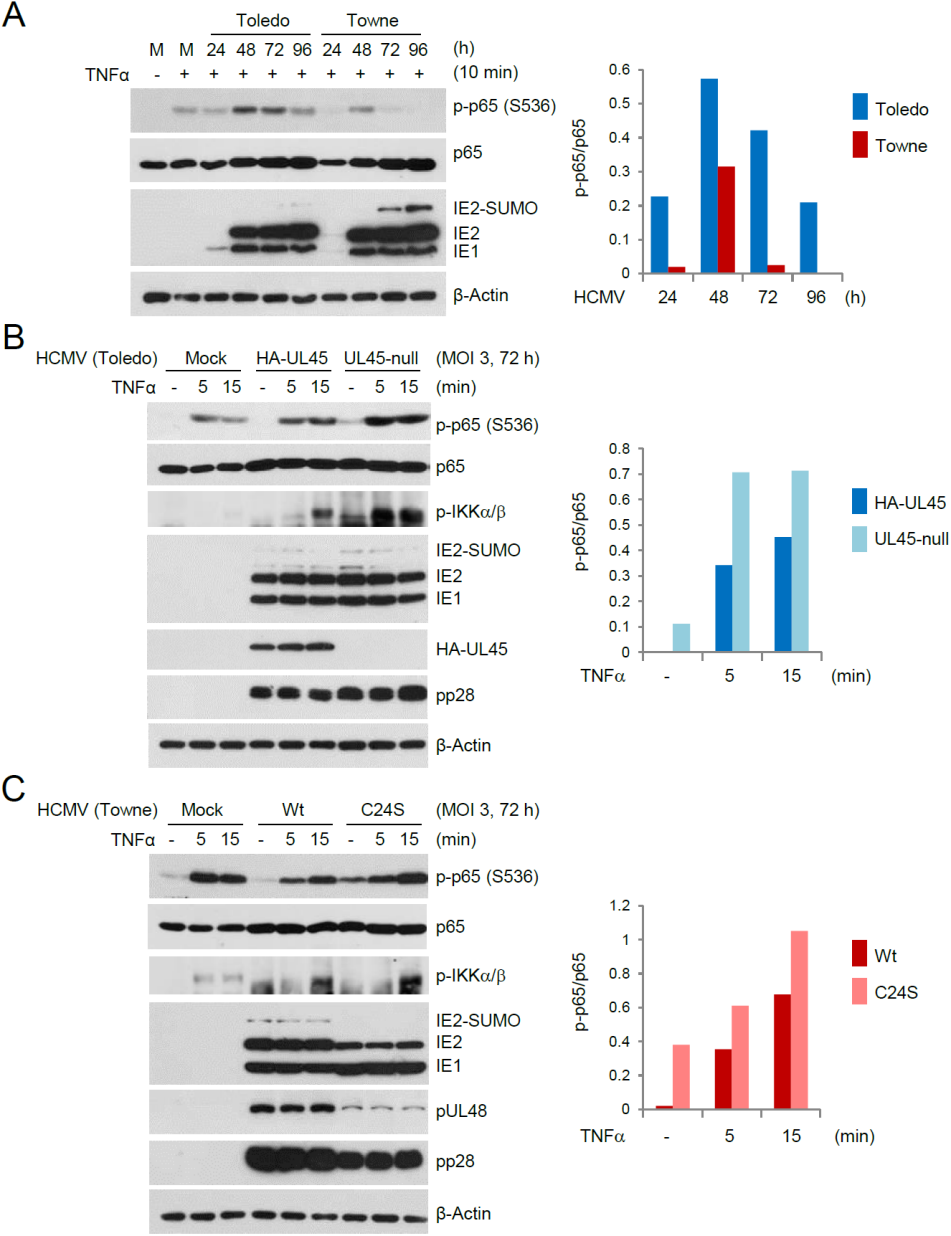
Inhibition of TNFα-induced NF-κB activation by UL45 and UL48 in the late stages of infection. (A) HF cells were mock infected or infected with HCMV Toledo or Towne viruses for the indicated time points at an MOI of 3. Cells were treated with TNFα (50 ng/ml) for 10 min. The total cell lysates were prepared and immunoblotting was performed with anti-p-p65(S536), p65, anti-IE1/IE2, or anti-β-actin antibody. (B and C) HF cells were mock infected or infected with HCMV Toledo virus (HA-UL45 or UL45-null) (B) or Towne virus [wild-type or UL48(C24S)] (C) for 72 h at an MOI of 3. Cells were treated with TNFα (50 ng/ml) for 5 or 15 min. Total cell lysates were prepared and immunoblotting was performed with antibodies for p-p65(S536), p65, p-IKKα/β, anti-IE1/IE2, HA-UL45, UL48, pp28, or β-actin. The levels of p65 and phosphorylated p65 were quantitated using ImageJ (NIH) and the changes of the ratio of phosphorylated p65 over p65 are shown as graphs.

RIP1 plays a key role in NF-κB signaling after TNFα treatment and HCMV inhibits NF-κB activation in the late stages of infection. Therefore, we investigated the effects of UL48 and UL45 on TNFα-induced NF-κB activation in the late stages of infection. We previously produced the DUB-defective UL48(C24S) mutant virus using the UL/b’ region-deleted Towne strain [14], while the UL45-null mutant in this study was produced in the Toledo strain. As a preliminary experiment, we compared the levels of NF-κB activation after TNFα treatment in cells infected with HCMV Toledo and Towne for different time periods. We found that phosphorylation of the NF-κB p65 subunit at serine residue 536 peaked 48 h after infection and began to decrease from 72 h in both Toledo and Towne virus-infected cells, while the phosphorylated p65 levels were higher during Toledo virus infection than during Towne virus infection (Fig 8A). The latter is consistent with the earlier reports demonstrating that the UL/b’ region in the Toledo virus and other clinical isolates sensitizes cells to TNFα signaling by upregulating cell surface expression of TNFR [39, 40].

Since UL45 and UL48 are expressed with early-late kinetics, their effects on TNFα-induced NF-κB activation were assessed 72 h after infection with wild-type or mutant viruses. In mock-infected cells, TNFα treatment for 5 or 15 min increased phosphorylation of p65 and IKKα/β. In Toledo virus infection, HA-UL45 virus less effectively upregulated TNFα-induced p65 and IKKα/β phosphorylation compared to UL45-null virus, demonstrating that UL45 expression inhibits TNFα-induced NF-κB activation (Fig 8B). In the case of Towne virus infection, wild-type virus resulted in less TNFα-induced p65 and IKKα/β phosphorylation compared to UL48(C24S) virus (Fig 8C). When we introduced the UL48(C24S) mutation in HA-UL45 Toledo virus, similar higher levels of p65 and IKKα/β phosphorylation were observed in UL48(C24S)-containing HA-UL45 virus compared to HA-UL45 virus (S5A and S5B Fig). In the experiments using UL48(C24S) mutant viruses, the levels of UL48(C24S) were lower than those of wild-type UL48. Therefore, it is not clear whether increased p65 and IKKα/β phosphorylation in mutant virus infection was due to the lack of DUB activity or due to the reduced protein level. However, since UL48(C24S) mutant protein that was expressed at a comparable level to wild-type protein largely lost the NF-κB repressing activity in reporter assays (see Figs 2D and S1A; Figs 4D and S1B), it is likely that the lack of UL48 DUB activity rather than the reduced level of UL48(C24S) largely contributed to the increase of TNFα-induced NF-κB activation. Supporting this idea, both HA-UL45 and RIP1 were still localized in the cVAC in HA-UL45/UL48(C24S) virus-infected cells (S5C Fig). Collectively, our analysis of UL45 and UL48 mutant viruses demonstrate that both UL45 and UL48 are involved in inhibition of TNFα-induced NF-κB activation in the late stages of infection.

### Cooperative effect of pUL48 and pUL45 on RIP1 deubiquitination

**Fig 9:**
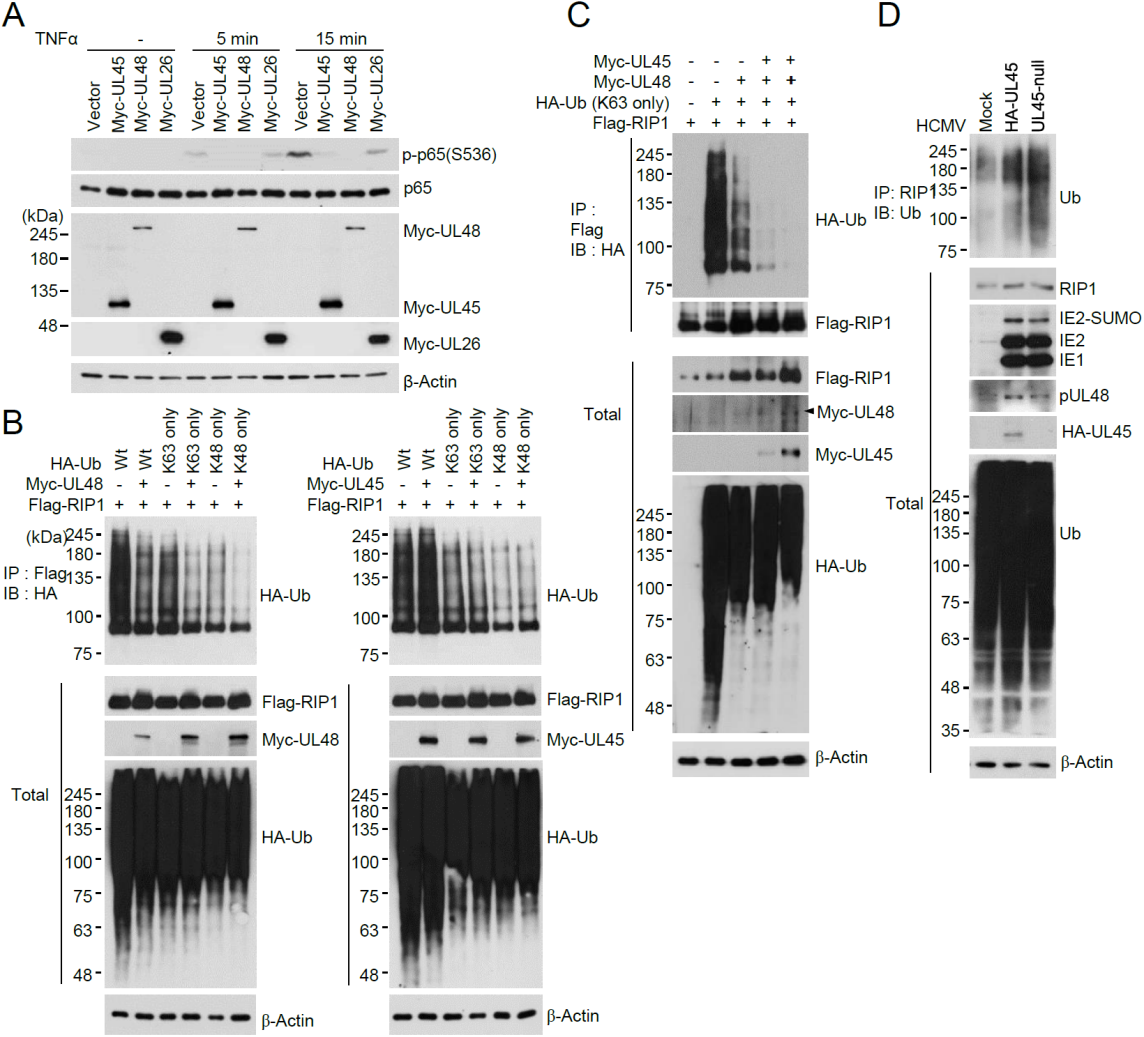
Cooperative effect of UL48 and UL45 on RIP1 deubiquitination. (A) HF cells were transfected with empty vector or plasmids expressing Myc-tagged UL45, UL48, or UL26 proteins via electroporation as indicated. At 24 h after transfection, cells were treated with TNFα (50 ng/ml) for 5 or 15 min. The total cell extracts were prepared and immunoblotting was performed with anti-p65, anti-p-p65(S536), anti-Myc, and anti-β-actin antibody. (B and C) 293T cells were co-transfected with plasmids expressing Flag-RIP1, HA-Ub construct, Myc-UL45, or Myc-UL48 as indicated. At 24 h after transfection, total cell lysates were immunoprecipitated with anti-Flag antibody, followed by immunoblotting with anti-HA or anti-Flag antibody. The total protein levels of Myc-UL45, Myc-UL48, Flag-RIP1, and HA-Ub conjugated proteins in cell lysates were also determined by immunoblotting. (D) HF cells were mock infected or infected with Toledo viruses (HA-UL45 or UL45-null) at an MOI of 2. At 72 h after infection, total cell lysates were immunoprecipitated with anti-RIP1 antibody and immunoblotting was performed with anti-ubiquitin antibody. The protein levels of RIP1, IE1, IE2, HA-UL45, and β-actin in total cell lysates were determined by immunoblotting.

The effect of UL45 and UL48 on TNFα-induced NF-κB activation was further investigated in HF cells co-transfected with UL45 and UL48 expression plasmids (Fig 9A). TNFα treatment increased phosphorylation of p65 in cells transfected with empty vector. However, TNFα-induced p65 phosphorylation was significantly impaired in cells transfected with UL45 or UL48. UL26, which was previously reported to antagonize NF-κB activation [24], also inhibited TNFα-induced p65 phosphorylation, but UL45 and UL48 did so more effectively, suggesting that UL45 and UL48 are potent inhibitors of NF-κB signaling.

To investigate the molecular mechanisms by which UL48 and UL45 inhibit TNFα-induced NF-κB activation, we assessed the effect of UL48 and UL45 expression on the levels of K63-linked polyubiquitination of RIP1. Co-transfection/ubiquitination assays were performed using plasmids expressing ubiquitin (K63 only) or ubiquitin (K48 only) in which all lysine (K) residues except K63 or K48 within ubiquitin are mutated to arginine to allow for the formation of only K63-linked or K48-linked ubiquitin chains. We found that UL48 inhibited RIP1 polyubiquitination through the K63 or K48 linkages, while UL45 did not affect RIP1 polyubiquitination (Fig 9B). When UL48 and UL45 were co-expressed, UL45 increased the activity of UL48 to cleave K63-linked polyubiquitin chains of RIP1 (Fig 9C). To address the role of UL45 in RIP1 ubiquitination in the context of viral infection, HF cells were infected with UL45-null or HA-UL45 virus and ubiquitination levels of endogenous RIP1 were examined. The results showed that the ubiquitination level of RIP1 was higher in UL45-null virus infection than in HA-UL45 virus infection (Fig 9D). These results demonstrate that UL48 inhibits TNFα-induced NF-κB activation through deubiquitination of RIP1 and that UL45 promotes this UL48 activity.

**Fig 10:**
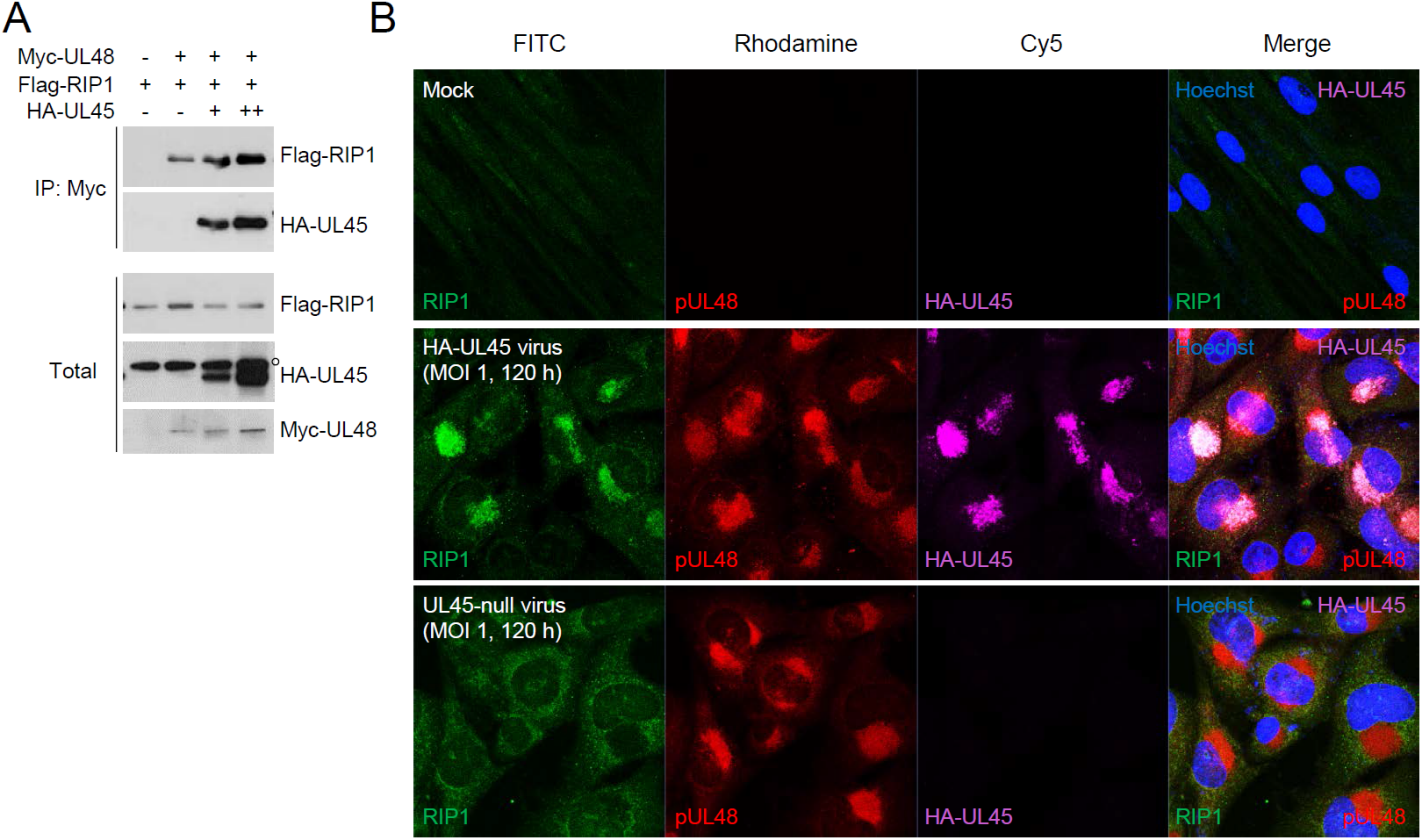
Promotion of RIP1 binding of UL48 by UL45. (A) 293T cells were co-transfected with plasmids expressing Myc-UL48, HA-UL45, or Flag-RIP1 as indicated. At 24 h after transfection, total cell lysates were immunoprecipitated with anti-Myc antibody and immunoblotting assays were performed with anti-HA or anti-Flag antibody. The total protein levels of Myc-UL48, HA-UL45, or Flag-RIP1 in cell lysates were also determined by immunoblotting. The open circle indicates non-specific bands. (B) HF cells were mock infected or infected with HCMV Toledo virus (HA-UL45 or UL45-null) for 120 h at an MOI of 1. Cells were fixed with cold methanol and triple-label confocal IFA was performed with anti-HA, anti-UL48, and anti-RIP1 antibodies. FITC-labeled anti-mouse IgG, rhodamine/Red X-coupled anti-rabbit IgG, and Cy5-conjugated anti-rat IgG antibodies were used for visualization. Hoechst stain was used to stain cell nuclei. Three side-by-side panels of signal-labeled images and a fourth panel with a merged image are shown.

We also found using CoIP assays that the interaction of UL48 with RIP1 in co-transfected cells was increased when UL45 was co-expressed, demonstrating that UL45 promotes the binding of UL48 with RIP1 (Fig 10A). Furthermore, when HF cells were infected with HA-UL45 virus, RIP1 was re-localized to the cVAC in all cells showing HA-UL45 in the cVAC; however, when cells were infected with UL45-null virus, this RIP1 re-localization to the cVAC was not observed (Fig 10B). Taken together, these results demonstrate that UL48 inhibits TNFα-induced NF-κB activation through deubiquitination of RIP1 and that UL45 promotes the binding of UL48 with RIP1 and re-localization of RIP1 to the UL48-containing cVAC.

### RIP1 targeting by viral DUB and R1 homolog is conserved in β-herpesviruses

Since the DUB-containing tegument proteins and R1 homologs are conserved in other herpesviruses, we further investigated whether RIP1 is targeted by DUB and R1 encoded by other human α- and γ-herpesviruses. The results of CoIP assays using HSV-1 proteins showed that UL36 (DUB) did not interact with RIP1, while UL39 (R1) bound to RIP1 as previously reported [41] (S6A and S6B Fig). In similar assays using KSHV proteins, neither ORF64 (DUB) nor ORF61 (R1) interacted with RIP1 (S6D and S6E Fig). Furthermore, the interaction between DUB and R1was not observed with HSV-1 and KSHV proteins in CoIP assays (S6C and S6F Fig). These results demonstrate that targeting RIP1 by viral DUB occurs in HCMV but not in HSV-1 and KSHV, whereas targeting RIP1 by viral R1 homolog is shared by both HCMV and HSV-1.

We further examined whether the RIP1 targeting by viral DUB and R1 homolog is conserved in β-herpesviruses. In CoIP assays with MCMV proteins, both M48 (DUB) and M45 (R1 homolog) interacted with mRIP1 (S7A and S7B Fig). Furthermore, M48 interacted with M45 (S7C Fig), suggesting that the activity of viral DUB and R1 homolog to target RIP1 and interact with each other may be preserved in β-herpesviruses (S7D Fig).

## Discussion

In this study, we identified RIP1 as a cellular substrate of the HCMV-encoded UL48 DUB. We showed that UL48 interacts with RIP1, cleaves its K48- and K63-linked polyubiquitin chains, and inhibits NF-κB activation induced by RIP1 overexpression or TNFα treatment. The inhibitory action of UL48 on NF-κB signaling largely required its DUB activity, although the catalytically inactive mutant protein still resulted in modest inhibition of NF-κB activation. Other herpesvirus-encoded DUBs, such as HSV-1 UL36, KSHV ORF64, and EBV BPLF1, have been shown to target TRAF3, RIG-I, and TRAF6, respectively [8–10]. Therefore, our findings support a notion that the herpesvirus-encoded DUBs target key regulators of host innate immune and inflammatory signaling. We found that MCMV M48 interacted with mRIP1, while HSV-1 UL36 and KSHV ORF64 did not interact with RIP1, and that the interaction between viral DUB and R1 homolog was observed with MCMV proteins but not with HSV-1 and KSHV proteins. These suggest that RIP1 targeting by viral DUB and the interaction between DUB and R1 homolog may be conserved in β-herpesviruses.

We also showed that UL45, an inactive viral homolog of cellular RNR R1, interacts with RIP1 and inhibits NF-κB activation mediated by RIP1 overexpression or TNFα treatment. In MCMV infection, M45, a homolog of UL45, was shown to interact with mRIP1 to inhibit mRIP1-mediated NF-κB activation or cell death. M45 interacted with mRIP1 in a RHIM domain-independent manner and inhibited TNFα-induced activation of NF-kB, p38 MAPK, and caspase-independent cell death [27]. A role of the RHIM-dependent interaction of M45 with RIP1 in suppressing cell death was also reported [31]. Furthermore, the M45 required RHIM-dependent interaction with DAI and RIP3 in order to block DAI-induced NF-κB activation [29] and RIP3-mediated necroptosis [32, 33]. Our data demonstrate that HCMV UL45 interacts with RIP1 independent of the RHIM since UL45, which does not contain an apparent RHIM, bound to a RHIM-deleted RIP1 mutant. Interestingly, unlike the R1 homologs encoded by α- and γ-herpesviruses, HCMV UL45 or other β-herpesvirus R1 homologs are catalytically inactive due to the absence of most residues known to have a catalytic role [42]. Why β-herpesviruses encode inactive R1 homologs is not fully understood. However, our findings, together with the above-mentioned previous works with MCMV M45, point to the possibility that the inactive R1 homologs of β-herpesviruses have been preserved to regulate RIP1-associated activities. M45 was also reported to target NEMO and induce its lysosomal degradation [28]. Whether UL45 also targets NEMO needs to be further investigated.

HCMV has been shown to suppress robust activation of NF-κB signaling during the late stages of infection and viral early and late proteins appear to be involved in this regulation [43–45]. Recently, UL26, an early protein, was found to inhibit NF-κB activation by attenuating IKK phosphorylation in TNFα-treated cells [24]. The results of the present study reveal that UL48 and UL45, expressed at delayed-early or late times after infection, are potent suppressors of NF-κB activation in the late stages of infection. In particular, the results of our transfection assays demonstrated that UL48 and UL45 more effectively suppress TNFα-induced NF-κB activation than UL26. Therefore, at least three viral proteins appear to downregulate NF-κB signaling in the late stages of infection. We observed that infection with the Toledo virus containing the UL/b’ region led to greater NF-κB activation after TNFα treatment than infection with the Towne virus lacking this region. It has been shown that the UL138 gene in the UL/b’ region is responsible for this greater sensitivity of Toledo by upregulating cell surface expression of TNFR and this UL138 activity may contribute to TNFα-induced reactivation of virus from latently infected cells [39, 40]. Notably, our results demonstrated that the lack of UL45 expression or inactivation of UL48 DUB still increased TNFα-induced NF-κB signaling in Toledo virus infection. HCMV appears to differentially regulate NF-κB activation during the early and late phases of infection and between lytic and latent infection. Therefore, it is not surprising at all that HCMV has been evolved to have several genes that positively or negatively regulate NF-κB activation [46].

We demonstrated that, although UL48 and UL45 are separately capable of targeting RIP1 and inhibiting RIP1-mediated NF-κB signaling, they have a cooperative effect on the regulation of NF-κB signaling. Although UL48 was previously shown to interact with UL45 in yeast two-hybrid interaction assays and co-transfection/CoIP assays [17, 35], we for the first time provide evidence that this interaction occurs in virus-infected cells. In co-transfection assays, UL45 increased the activity of UL48 to cleave K63-linked polyubiquitination of RIP1 and interact with RIP1. More importantly, the ubiquitination level of endogenous RIP1 was higher in the late stages of infection during UL45-null virus infection compared to infection with HA-UL45 virus. One possible mechanism for the cooperative activity of UL45 is that UL45 binding to RIP1 alters RIP1 conformation or localization, converting it to a better substrate for UL48 DUB activity. Indeed, we observed that UL45 promoted the RIP1 binging by UL48 in co-transfected cells and re-localization of RIP1 to the UL48-containing cVAC in virus-infected cells. It would also be intriguing to test whether UL45 targets other UL48 substrates and promotes UL48’s DUB activity toward them.

Accumulation of deubiquitinated RIP1 in cells is a key step for the switch from pro-survival to pro-apoptotic function of RIP1. Our observation that UL48 and UL45 cooperatively increase the level of deubiquitinated RIP1 raises a question whether the expression of UL48 and UL45 sensitizes cells to apoptosis. We investigated this possibility by comparing anti-Fas/cycloheximide-induced cleavage of poly (ADP-ribose) polymerase (PARP) between wild-type and UL48(C24S) mutant virus-infected HF cells. However, we did not observe higher level of PARP cleavage by wild type virus than mutant virus. We reason this to the expression of strong viral inhibitor of caspase-8-induced apoptosis (vICA) [47, 48] that works at downstream of RIP1.

Both UL48 and UL45 are viral tegument proteins that are present within virions and delivered into host cells upon virus entry. Therefore, these proteins may also regulate NF-κB signaling immediately after virus entry. The interaction of these proteins may also play a role in the nuclear egress of the capsid or virion maturation in the cytoplasm, but this awaits further investigation.

## Materials and methods

### Cell culture and virus

Primary human foreskin fibroblast (HF) (American Type Culture Collection; ATCC PCS-201-010) and human embryonic kidney (HEK) 293T cells (ATCC) were grown in Dulbecco’s modified Eagle’s medium (DMEM) supplemented with 10% fetal bovine serum, penicillin (100 U/ml), and streptomycin (100 μg/ml) in a 5% CO_2_ humidified incubator at 37°C. Stocks of the Towne wild-type and UL48(C24S) mutant viruses were prepared as described previously [14]. To produce UV-inactivated HCMV (UV-HCMV), the virus stock was irradiated with UV light three times at 0.72 J/cm^2^ using a CL-1000 cross-linker (UVP). For infection experiments, HF cells were infected with virus at specified multiplicities of infection (MOI). At the indicated times, the growth medium was collected and combined with lysates prepared from the cell layer by freezing and thawing three times, clarified by centrifugation, and stored at -70°C until assayed for infectivity.

### Plasmids

The wild-type UL45 DNA (Towne strain) was cloned into pENTR vectors (Invitrogen). Plasmid expressing Myc-tagged UL45 was produced by transferring the DNA to a pCS3-MT (with a 6Myc tag)-based destination vector using LR Clonase (Invitrogen). Plasmids expressing Myc-tagged UL48 (wild-type or C24S mutant) were described previously [14]. Plasmid expressing hemagglutinin (HA)-tagged UL48 was generated on a pSG5 background using Gateway technology as previously described [17]. Plasmid for flag-tagged RIP1 was provided by Jaehwan Song (Yonsei University). The wild-type RIP1 DNA was cloned into pENTR vectors (Invitrogen). Plasmid for HA-tagged RIP1 was produced by transferring the DNA to a pSG5-based destination vector using LR Clonase (Invitrogen). Plasmids encoding HA-tagged ΔKD, ΔID, ΔDD, ΔRHIM, and K377R mutant RIP1were generated by PCR in the same background. Plasmids expressing HSV-1 UL36 (pUL36-EGFP-N2) and KSHV ORF64 (pcDEF-Flag-ORF64) were provided by Prashant Desai (Johns Hopkins University) and Kyung-Soo Inn (Kyung Hee University), respectively. The HSV-1 UL39 and KSHV ORF61 genes were PCR cloned from viral DNAs into pENTR vector and plasmids expressing myc-tagged proteins were produced on a pCS3-MT-based destination vector using LR Clonase. pENTR vectors containing mouse RIP1 and MCMV M45 were produced by PCR from pCMV2-Flag-mRIP1 and pcDNA-M45-HA, respectively, that were provided by Wolfram Brune (Leibniz Institute for Experimental Virology). pENTR vector containing MCMV M48 was also produced by PCR from pEGFP-N1-M48 that was gifted by Jason W. Upton (University of Texas at Austin). pSG5 plasmids expressing HA-mRIP1 and HA-M45 and pCS3-MT plasmids expressing Myc-M45 and Myc-M48 were produced by transferring the DNAs into appropriate destination vectors using LR clonase. Plasmids for HA-Ub and CrmA were provided by Hongtae Kim (Sungkyunkwan University) and Kyeong Sook Choi (Ajou University), respectively.

### Antibodies

Mouse monoclonal antibody (MAb) 810R, which detects epitopes present in both IE1 and IE2, was obtained from Millipore. Chicken egg yolk anti-UL45 IgY antibodies were gifted from Andrea Gallina (University of Milano). Rabbit polyclonal antibody (PAb) raised against the synthetic peptide corresponding to UL48 residues 278 to 295 (anti-UL48-N) was provided by Wade Gibson (Johns Hopkins University School of Medicine) [14]. Mouse MAbs against p52 (encoded by UL44) and pp28 (UL99) were purchased from Advanced Biotechnologies. The anti-pp65 (UL83) mouse MAb was purchased from Virusys. The anti-HA rat MAb (3F10) either conjugated with peroxidase or labeled with fluorescein isothiocyanate (FITC), anti-HA mouse MAb (12CA5), anti-HA rat MAb (3F10), and anti-Myc mouse MAb (9E10) conjugated with peroxidase were purchased from Roche. The anti-flag mouse MAb M2 was obtained from Sigma. Mouse MAbs for RIP1 (C-12) and p65 NF-κB subunit were purchased from Santa Cruz Biotechnology and Ab Frontier, respectively. The anti-phospho-p65(S536) and anti-phospho-IKKα/β rabbit MAbs were obtained from Cell Signaling Technology. Mouse MAbs for α-tubulin and β-actin were purchased from Sigma. Rabbit PAbs against NF-κB p50 and HDAC2 were purchased from Upstate and Zymed, respectively. Secondary antibodies such as FITC-labeled donkey anti-mouse IgG, Rhodamine Red-X-conjugated donkey anti-mouse IgG, Rhodamine Red-X-conjugated donkey anti-rabbit IgG, and Cy5-conjugated donkey anti-rat IgG were obtained from Jackson ImmunoResearch Laboratories, Inc.

### Bacmid mutagenesis

The HCMV (Toledo strain) bacterial artificial chromosome (BAC) (Toledo-BAC) clone was kindly provided by Hua Zhu (Rutgers-New Jersey Medical School). The Toledo BAC clones encoding the UL45stop (UL45-null) and HA-tagged UL45 (HA-UL45) genes were generated using a counter-selection BAC modification kit (Gene Bridges). Briefly, the rpsL-neo cassette DNA was PCR-amplified using LMV1708/1731 primers containing homology arms consisting of 50 nucleotides upstream and downstream of the target region plus 24 nucleotides homologous to the rpsL-neo cassette. The amplified rpsL-neo fragments with homology arms were purified and introduced into *E*. *coli* DH10B containing the wild-type Toledo-BAC clone for recombination by electroporation using a Gene Pulser II (Bio-Rad). The intermediate Toledo-BAC constructs containing the rpsL-neo cassette were selected on Luria broth (LB) plates containing kanamycin. Next, the UL45stop fragments for replacing the rpsL-neo cassette were amplified by PCR using LMV1732/1733 primers. The amplified fragments were recombined into the Toledo-BAC DNA containing the rpsL-neo cassette, and the UL45-null Toledo-BAC was selected on LB plates containing streptomycin. The HA-UL45 Toledo-BAC clone was also generated from the UL45-null Toledo-BAC by the same method using LMV1494/1495/1733 primers. LMV primers used for bacmid mutagenesis were as follows: LMV1708, 5’-TCACTTTATTGAAATCTACCTGATTTCTTTGTTATTTTCCTCGTAAACTTGGCCTGGTGATGATGGCGGGATCG-3’; LMV1731, 5’- GCCGTCGGGAGACGGCGACTCGGGACGCCAACTGACGACGCCGCCACCACTCAGAAGAACTCGTCAAGAAGGCG-3’; LMV1732, 5’- TCACTTTATTGAAATCTACCTGATTTCTTTGTTATTTTCCTCGTAAACTTATGAATCCGGCTGACGCGGA-3’; LMV1733, 5’-GCCGTCGGGAGACGGCGACTCGGGACGCCA-3’; LMV1494, 5’-ATGTACCCATACGATGTTCCAGATTACGCTATGAATCCGGCTGACGCGGACGAGGAACAGCGGGTGTCCT-3’; and LMV1495 5’-TCACTTTATTGAAATCTACCTGATTTCTTTGTTATTTTCCTCGTAAACTTATGTACCCATACGATGTTCC-3’.

### Electroporation and production of virus stocks

Electroporation was used to introduce the Toledo-BAC DNA into HF cells. For each reaction, HF cells (2 x 10^6^) in 400 μl of resuspension buffer were mixed with 5 μg of Toledo-BAC DNA, 2 μg of plasmid pCMV71 encoding pp71 to enhance activation of the major immediate-early (MIE) promoter, and 1 μg of plasmid pEGFP-C1 to monitor electroporation efficiency. Electroporation was done at 1300 V for 30 ms using a Microporator MP-100 (Digital Bio Technology), and the cells were transferred to T-25 plates. When the surviving cells reached confluence, cells were transferred at a dilution of 1:2 into new flasks.

### Transient DNA transfection

293T cells were transfected via polyethylenimine (PEI) (Sigma). A mixture of plasmid DNA and serum-free media was mixed with PEI. The volume of PEI used was based on a 3:1 ratio of PEI (μg):total DNA (μg). The mixture was kept at room temperature for 20 min and then added dropwise to cells. HF cells were transfected via OmicsFect (Omics Bio) or via electroporation. Electroporation was performed at 1300 V for 30 ms using a Microporator MP-100 (Digital Bio). The transfection efficiency of HF cells using electroporation under these conditions was about 50 to 60% (S1C Fig).

### Coimmunoprecipitation (CoIP) assay

Cell lysates were harvested and prepared by sonication in 1 ml CoIP buffer (50 mM Tris-Cl [pH 7.4], 50 mM NaF, 5 mM sodium phosphate, 0.1% Triton X-100, containing protease inhibitors [Sigma]) using a Vibra cell microtip probe (Sonics and Materials) for 10 sec (pulse on: l s, pulse off: 0.5 s). The clarified cell lysates were incubated for 16 h with the appropriate antibody at 4°C. A total of 30 μl of 50% slurry of protein A- and G-Sepharose (Amersham) was added. After incubation for 2 h at 4°C, the mixture were pelleted and washed several times with CoIP buffer. Each sample was analyzed by SDS-PAGE and immunoblotting with the appropriate antibody.

### Immunoblot analysis

The DNA-transfected cells or virus-infected cells were harvested with phosphate-buffered saline (PBS) and total cell extracts were prepared by boiling the cell pellets in sodium dodecyl sulfate (SDS) loading buffer. Equal amounts of the clarified cell extracts were separated by SDS-PAGE, and then transferred onto a nitrocellulose membrane (GE Healthcare Life Sciences) or PVDF membrane (Millipore). The membrane was blocked for 1 h in PBST (PBS plus 0.1% Tween 20 [Sigma]) containing 5% skim milk and then washed with PBST. After incubation with the appropriate antibodies, the proteins were visualized by standard procedures using an enhanced chemiluminescence system (Roche) and x-ray film (Kodak).

### Gel filtration chromatography

Cell extracts were prepared and loaded onto a Superose6 10/300 GL column (GE Healthcare) pre-equilibrated with CoIP buffer. The proteins were eluted at 0.5 ml/min. Each fraction (15 μl) was analyzed by immunoblotting with antibodies for RIP1, HA-UL45, and UL48. Apparent molecular mass was evaluated after column calibration with standard proteins [thyroglobulin (669 kDa), ferritin (440 kDa), aldolase (158 kDa), conalbumin (75 kDa), and ovalumin (44 kDa)] in the Gel Filtration Calibration Kit (GE Healthcare).

### Indirect immunofluorescence assay (IFA)

Cells were fixed in cold methanol for 5 min and rehydrated in cold PBS. Cells were first incubated for 30 min with human γ-globulin (Sigma) (2 mg per ml) at 37°C to block non-specific binding of antibodies to HCMV-induced Fc receptors [49]. They were then incubated for 1 h with primary antibodies in PBS at 37°C and then for 1 h with appropriate secondary antibodies labeled with FITC, Rhodamine Red-X, or Cy5. Antibodies were incubated together for triple labeling. The slides were examined and photographed with a Carl Zeiss LSM710Meta confocal microscope system.

### Infectious center assay

Diluted samples were used to inoculate a monolayer of HF cells (1 x 10^5^) in a 24-well plate. At 24 h post-infection, cells were fixed with 500 μl of cold methanol for 10 min. Cells were then washed three times in phosphate-buffered saline (PBS), incubated with anti-IE1 rabbit PAb in PBS at 37°C for 1 h, followed by incubation with phosphatase-conjugated anti-rabbit IgG antibody in PBS at 37°C for 1 h. Finally, the cells were gently washed in PBS and treated with 200 μl of developing solution (nitroblue tetrazolium/5-bromo-4-chloro-3-indolylphosphate) at room temperature for 1 h according to the manufacturer's instructions. The IE1-positive cells were counted in at least three to five separate fields per well under a light microscope (200X magnification).

### Luciferase reporter assay

Cells were collected and lysed by three freeze-thaw steps in 200 μl of 0.25 M Tris-HCl (pH 7.9) plus 1 mM dithiothreitol. Cell extracts were clarified in a microcentrifuge, and 20 μl of each extract was incubated with 350 μl of reaction buffer A (25 mM glycyl-glycine [pH 7.8], 5 mM ATP [pH 7.5], 4 mM EGTA [pH 8.0], 15 mM MgSO_4_) and then mixed with 100 μl of 0.25 mM luciferin (Sigma) in reaction buffer A. A TD-20/20 luminometer (Turner Designs) was used for a 10-s assay of the photons produced (measured in relative light units).

### β-galactosidase reporter assay

Cells were collected and lysed by three freeze-thaw steps in 200 μl of 0.25 M Tris-HCl (pH 7.9) plus 1 mM dithiothreitol. Cell extracts were clarified in a microcentrifuge. Each reaction mix was prepared in a 96-well plate including 20 μl of extract plus 100 μl of Z-buffer (0.06 M Na_2_HPO_4_, 0.04 M NaH_2_PO_4_, 0.01 M KCl, 0.001 M MgSO_4_, 0.05 M β-mercaptoethanol) plus 20 μl of o-nitrophenyl-β-D-galactopyranoside (ONPG [Sigma]; 4 mg/ml in sterile water). Reactions were incubated at 37°C until the yellow color developed. The reaction was stopped by adding 50 μl of 1 M Na_2_CO_3_. The β-galactosidase absorbance of each reaction was read at 420 nm.

### Ubiquitination assays

293T cells were co-transfected with plasmids expressing target or effector proteins and plasmids expressing HA-ubiquitin. At 24 h after transfection, cell pellets were re-suspended with 2% SDS lysis buffer containing protease inhibitors (Sigma) and boiled for 10 min. Cell lysates were diluted ten-fold with CoIP buffer and sonicated using a Vibra cell microtip probe. The clarified cell lysates were incubated with 30 μl of 50% slurry of anti-Flag M2 affinity gel (Sigma) for 16 h at 4°C. The mixture was pelleted and washed several times with CoIP buffer. The bound proteins were boiled and analyzed by SDS-PAGE and immunoblotting with the appropriate antibody. To detect endogenous RIP1 ubiquitination in virus-infected cells, the mock-infected or virus-infected cells were treated with 5 mM N-ethylmaleimide (NEM) for 30 min before they were harvested. Cells were lysed in PBS containing 1% SDS and 5 mM NEM and then boiled for 10 min. Cell lysates were sonicated and immunoprecipitated with anti-RIP1 antibody (BD Biosciences). For immunoblotting, proteins were transferred to PVDF membranes and denatured using 6 M guanidine-HCl containing 20 mM Tris-HCl (pH 7.5), 1 mM PMSF, and 0.5 mM β-mercaptoethanol for 30 min at 4°C. Ubiquitinated RIP1 was identified by HRP-conjugated anti-ubiquitin antibody (Biomol).

### Statistical analysis

Samples were compared using Student’s *t*-test, and p-values <0.05 (*) <0.01 (**) and <0.001 (***) are indicated in the figures.

## Supporting information

S1 Fig The expression levels of transfected proteins in reporter assays. (A and B) The expression levels of proteins in transfected 293T cells in Figs 2D and 4D are shown in (A) and (B), respectively. (C) Images showing transfection efficiency of HF with a GFP-expressing plasmid using electroporation. Electroporation was performed using a Microporator MP-100 (Digital Bio Technology) at 1300 V for 20 ms.
(TIF)

S2 Fig Generation of UL45 deletion mutant and HA-UL45 recombinant viruses. (A) Strategy for the HCMV UL45 gene mutation. Three viruses were prepared from a Toledo HCMV bacmid: wild-type virus, UL45 mutant virus with three stop codons in the N-terminal region (UL45-null), and a recombinant virus encoding UL45 with an N-terminal HA-tag (HA-UL45). The four inserted nucleotides for UL45-null virus and 30 nucleotides for HA-UL45 virus are indicated in italics. (B) The Toledo-BAC clone encoding UL45stop and HA-UL45 was produced using a counter-selection BAC modification kit (Gene Bridges) (see Materials and Methods). (C) Wild-type, UL45-null, and HA-UL45 bacmids were digested with EcoRV and BamH1 and subjected to agarose gel electrophoresis. No apparent alteration of restriction fragment patterns was found in UL45-null and HA-UL45 bacmids. (D) HF cells were mock-infected or infected with wild-type, UL45-null, or HA-UL45 viruses at an MOI of 2. At 5 days after infection, total cell lysates were prepared and immunoblotted for HA-UL45, UL45, IE1, IE2, and β-actin. (E) HF cells were mock-infected or infected with wild-type or HA-UL45 viruses at an MOI of 1. Total cell lysates were prepared at indicated time points and immunoblotting was performed as in (D).
(TIF)

S3 Fig A control IFA without primary antibody treatment. HF cells were mock-infected or infected with HA-UL45 Toledo virus for 96 h at an MOI of 1 as in Fig 7. Cells were fixed with cold methanol and then incubated with γ-globulin as a blocking agent, followed by incubation with secondary antibodies (FITC-labeled anti-mouse IgG, Rhodamine/Red X-coupled anti-rabbit IgG, and Cy5-conjugated anti-rat IgG antibodies). Hoechst stain was used to stain cell nuclei. The images were obtained by confocal microscopy. Three side-by-side panels of signal-labeled images and a fourth panel with a merged image (including DNA staining) are shown.
(TIF)

S4 Fig Double-label merge images demonstrating colocalization among RIP1, UL48, and HA-UL45 in HA-UL45 virus-infected cells. Enlarged double-label merge images were shown for RIP1 and pUL48, RIP1 and HA-UL45, and pUL48 and HA-UL45 (with nuclear staining) from Fig 7C.
(TIF)

S5 Fig Effect of the UL48(C24S) mutation on TNFα-induced NF-κB activation in the late stages of Toledo virus infection. (A) The HCMV (Toledo) bacmid containing the UL48(C24S) gene was generated from the HA-UL45 bacmid using a counter-selection BAC modification kit (Gene Bridges) as in S2A Fig. First, the rpsL-neo cassette DNA was PCR-amplified using LMV2126/2127 primers containing homology arms and introduced into *E*. *coli* DH10B containing the HA-UL45 Toledo-BAC by electroporation to produce the rpsL-neo cassette-containing intermediate BAC constructs. Second, the UL48(C24S) fragments for replacing the rpsL-neo cassette were amplified by PCR using LMV2128/2129 primers and introduced into the rpsL-neo cassette-containing intermediates. The HA-UL45/UL48(C24S) Toledo-BAC clone was selected on LB plates containing streptomycin. LMV primers used for bacmid mutagenesis were as follows: LMV2126, 5’-GCTGCCACCAGGGCGACATCGCCCGCTTTGGAGCGCGAGCGGGCAATCAAGGCCTGGTGATGATGGCGGGATCG-3’; LMV2127, 5’- CTCGTTCCACCCAGGTGCAAGGCGTGTAGGAACATGATGCCGTTGCAGACTCAGAAGAACTCGTCAAGAAGGCG-3’; LMV2128, 5’- GCTGCCACCAGGGCGACATCGCCCG-3’; and LMV2129, 5’-CTCGTTCCACCCAGGTGCAAGGCGT-3’. (B) HF cells were mock-infected or infected with HA-UL45 virus at an MOI of 2 or with HA-UL45/UL48(C24S) virus at an MOI of 2 or 4 for 72 h. Cells were treated with TNFα (50 ng/ml) for 5 or 15 min. Total cell lysates were prepared and immunoblotting was performed with antibodies for p-p65(S536), p65, p-IKKα/β, anti-IE1/IE2, HA-UL45, UL48, pp28, or β-actin. Non-specific bands were denoted by open circles. The levels of p65 and phosphorylated p65 were quantitated by counting using ImageJ (NIH) and the changes of the ratio of phosphorylated p65 over p65 are shown as a graph. (C) HF cells were infected with HA-UL45 or HA-UL45/UL48(C24S) viruses for 96 h at an MOI of 1 and triple-label IFA was performed as in Fig 7C.
(TIF)

S6 Fig Interaction of DUB and R1 encoded by HSV-1 and KSHV with RIP1. 293T cells were co-transfected with plasmid expressing HA-RIP1 and plasmids expressing HSV-1 proteins (UL36-EGFP and Myc-UL39) (A to C) or plasmids expressing KSHV proteins (Flag-ORF64, or Myc-ORF61 (D to F) as indicated. At 24 h after transfection, total cell lysates were immunoprecipitated with anti-HA or anti-Myc antibody and immunoblotting assays were performed as indicated. The protein levels in total cell lysates were also determined by immunoblotting.
(TIF)

S7 Fig Comparison of the interaction of viral DUB and R1 with RIP1 between MCMV and HCMV. (A and B) 293T cells were co-transfected with plasmid expressing HA-RIP1 (mRIP1 or hRIP1) and plasmid expressing Myc-tagged viral DUB (M48 or UL48) (A) or plasmid expressing Myc-tagged viral R1 (M45 or UL45) (B), as indicated. At 24 h after transfection, total cell lysates were immunoprecipitated with anti-Myc antibody and immunoblotting assays were performed as indicated. The protein levels in total cell lysates were also determined by immunoblotting. (C) 293T cells were co-transfected with plasmids expressing Myc-tagged viral DUB (M48 or UL48) and HA-tagged viral R1 (M45 or UL45) as indicated. CoIP assays were performed as in (A). (D) Summary of the activity of viral DUB and R1 homolog to target RIP1 and interact with each other in different herpesviruses.
(TIF)
