# Supplementary figures and images for: Cooperative inhibition of RIP1-mediated NF-κB signaling by cytomegalovirus-encoded deubiquitinase and inactive homolog of cellular ribonucleotide reductase large subunit
Source: PLoS Pathog. 2017 Jun 1;13(6):e1006423. doi: 10.1371/journal.ppat.1006423 (PMC5469499; doi:10.1371/journal.ppat.1006423)

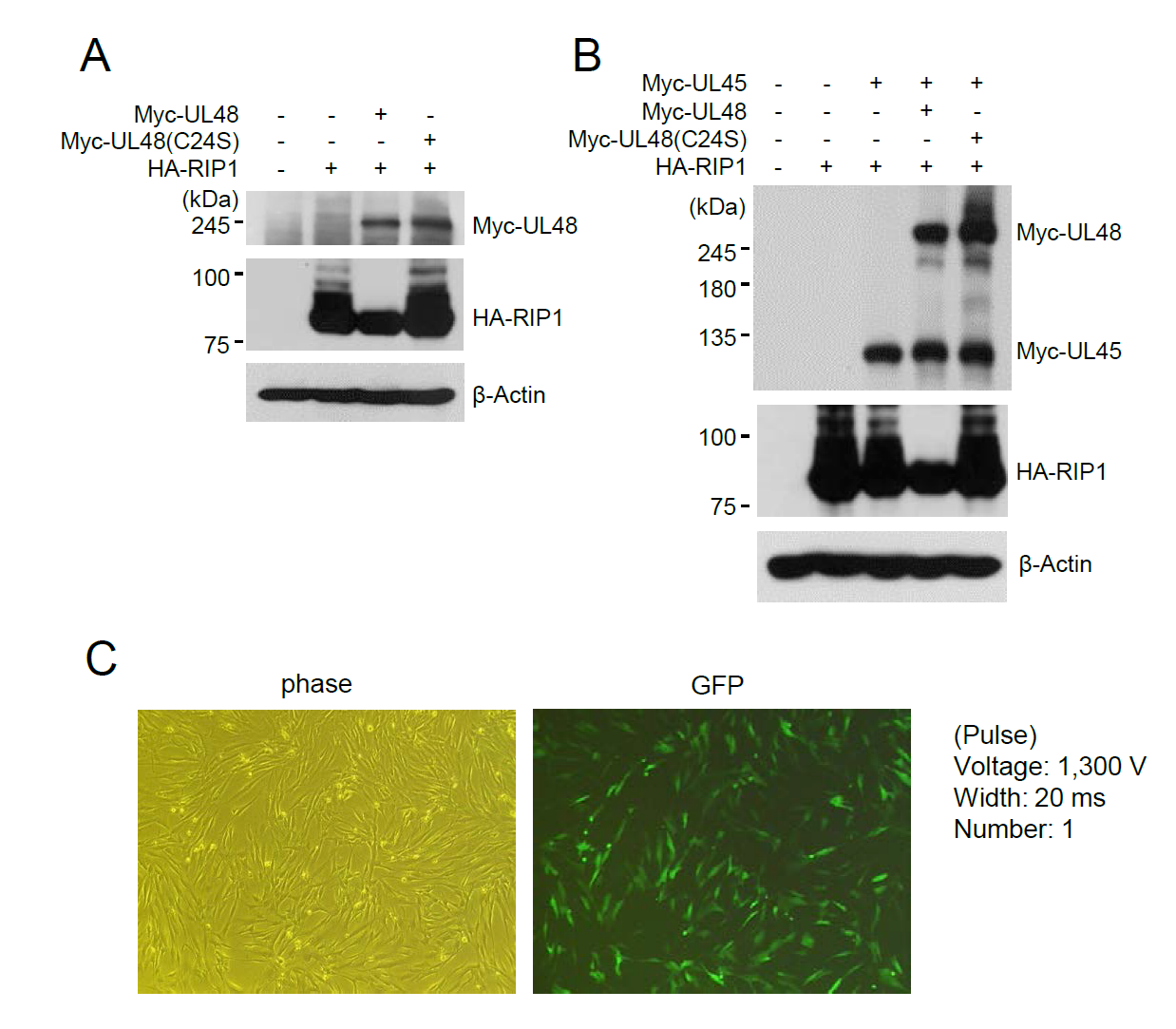

Supplement: S1 Fig — (A and B) The expression levels of proteins in transfected 293T cells in Figs 2D and 4D are shown in (A) and (B), respectively. (C) Images showing transfection efficiency of HF with a GFP-expressing plasmid using electroporation. Electroporation was performed using a Microporator MP-100 (Digital Bio Technology) at 1300 V for 20 ms. (TIF) [file ppat.1006423.s001.tif]

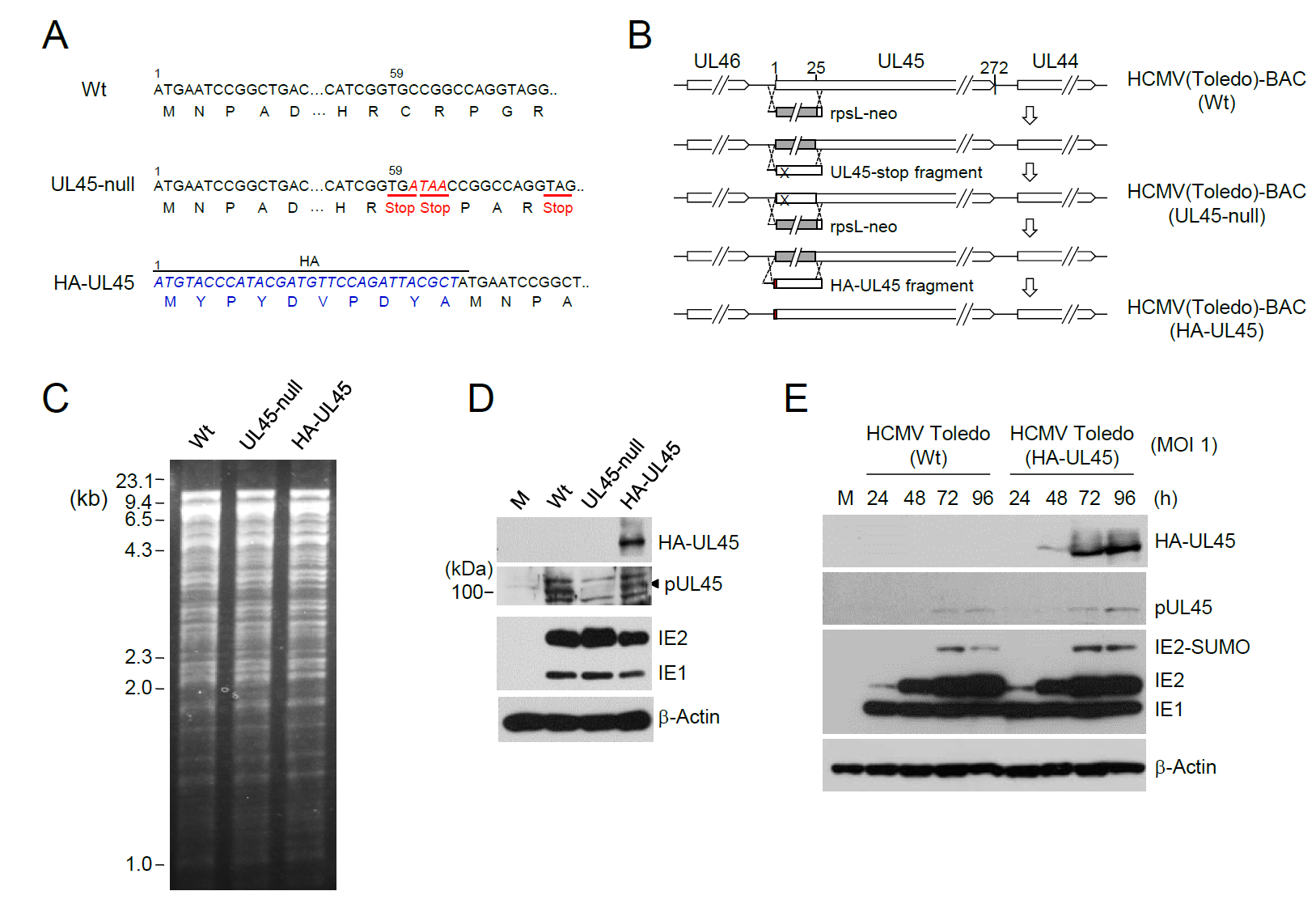

Supplement: S2 Fig — (A) Strategy for the HCMV UL45 gene mutation. Three viruses were prepared from a Toledo HCMV bacmid: wild-type virus, UL45 mutant virus with three stop codons in the N-terminal region (UL45-null), and a recombinant virus encoding UL45 with an N-terminal HA-tag (HA-UL45). The four inserted nucleotides for UL45-null virus and 30 nucleotides for HA-UL45 virus are indicated in italics. (B) The Toledo-BAC clone encoding UL45stop and HA-UL45 was produced using a counter-selection BAC modification kit (Gene Bridges) (see Materials and Methods). (C) Wild-type, UL45-null, and HA-UL45 bacmids were digested with EcoRV and BamH1 and subjected to agarose gel electrophoresis. No apparent alteration of restriction fragment patterns was found in UL45-null and HA-UL45 bacmids. (D) HF cells were mock-infected or infected with wild-type, UL45-null, or HA-UL45 viruses at an MOI of 2. At 5 days after infection, total cell lysates were prepared and immunoblotted for HA-UL45, UL45, IE1, IE2, and β-actin. (E) HF cells were mock-infected or infected with wild-type or HA-UL45 viruses at an MOI of 1. Total cell lysates were prepared at indicated time points and immunoblotting was performed as in (D). (TIF) [file ppat.1006423.s002.tif]

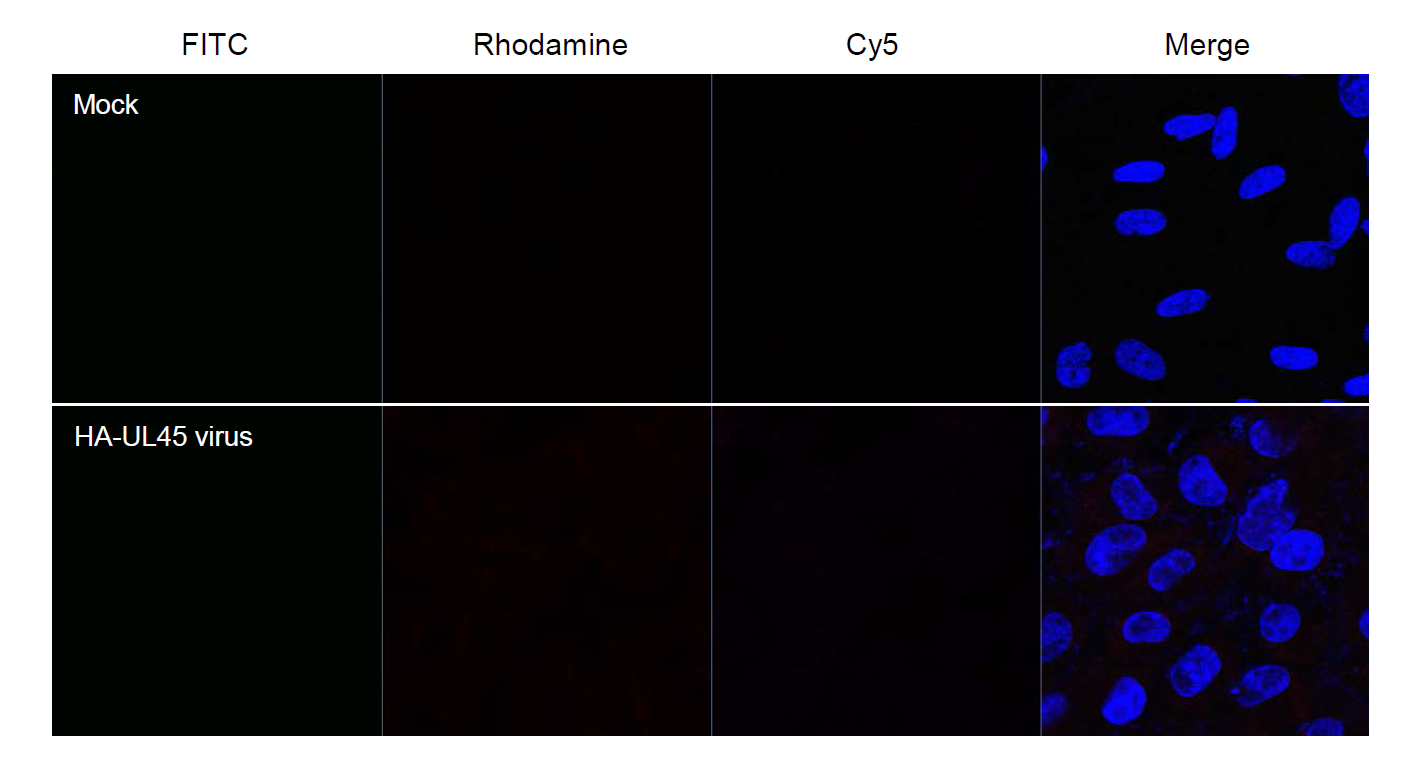

Supplement: S3 Fig — HF cells were mock-infected or infected with HA-UL45 Toledo virus for 96 h at an MOI of 1 as in Fig 7. Cells were fixed with cold methanol and then incubated with γ-globulin as a blocking agent, followed by incubation with secondary antibodies (FITC-labeled anti-mouse IgG, Rhodamine/Red X-coupled anti-rabbit IgG, and Cy5-conjugated anti-rat IgG antibodies). Hoechst stain was used to stain cell nuclei. The images were obtained by confocal microscopy. Three side-by-side panels of signal-labeled images and a fourth panel with a merged image (including DNA staining) are shown. (TIF) [file ppat.1006423.s003.tif]

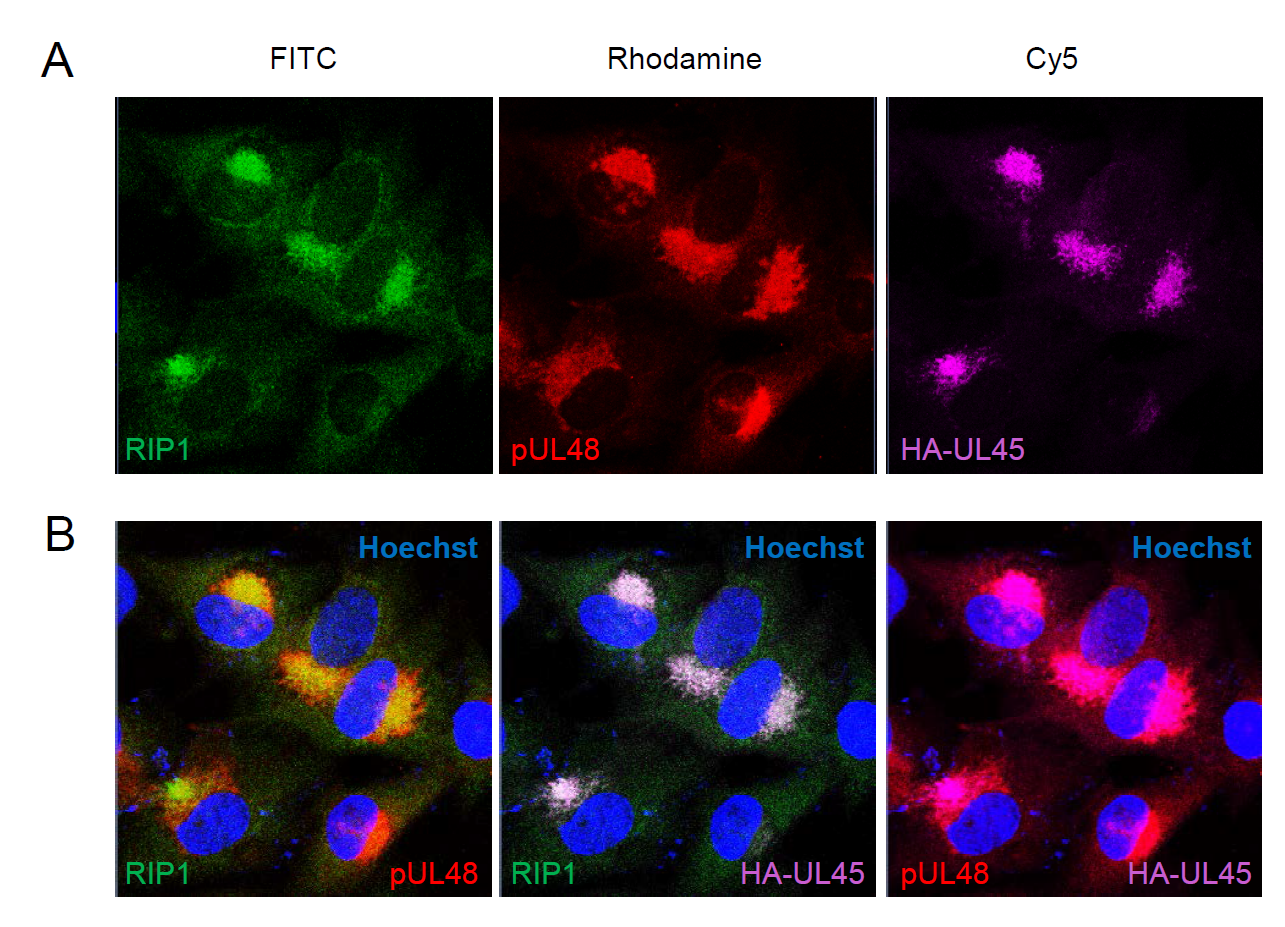

Supplement: S4 Fig — Enlarged double-label merge images were shown for RIP1 and pUL48, RIP1 and HA-UL45, and pUL48 and HA-UL45 (with nuclear staining) from Fig 7C. (TIF) [file ppat.1006423.s004.tif]

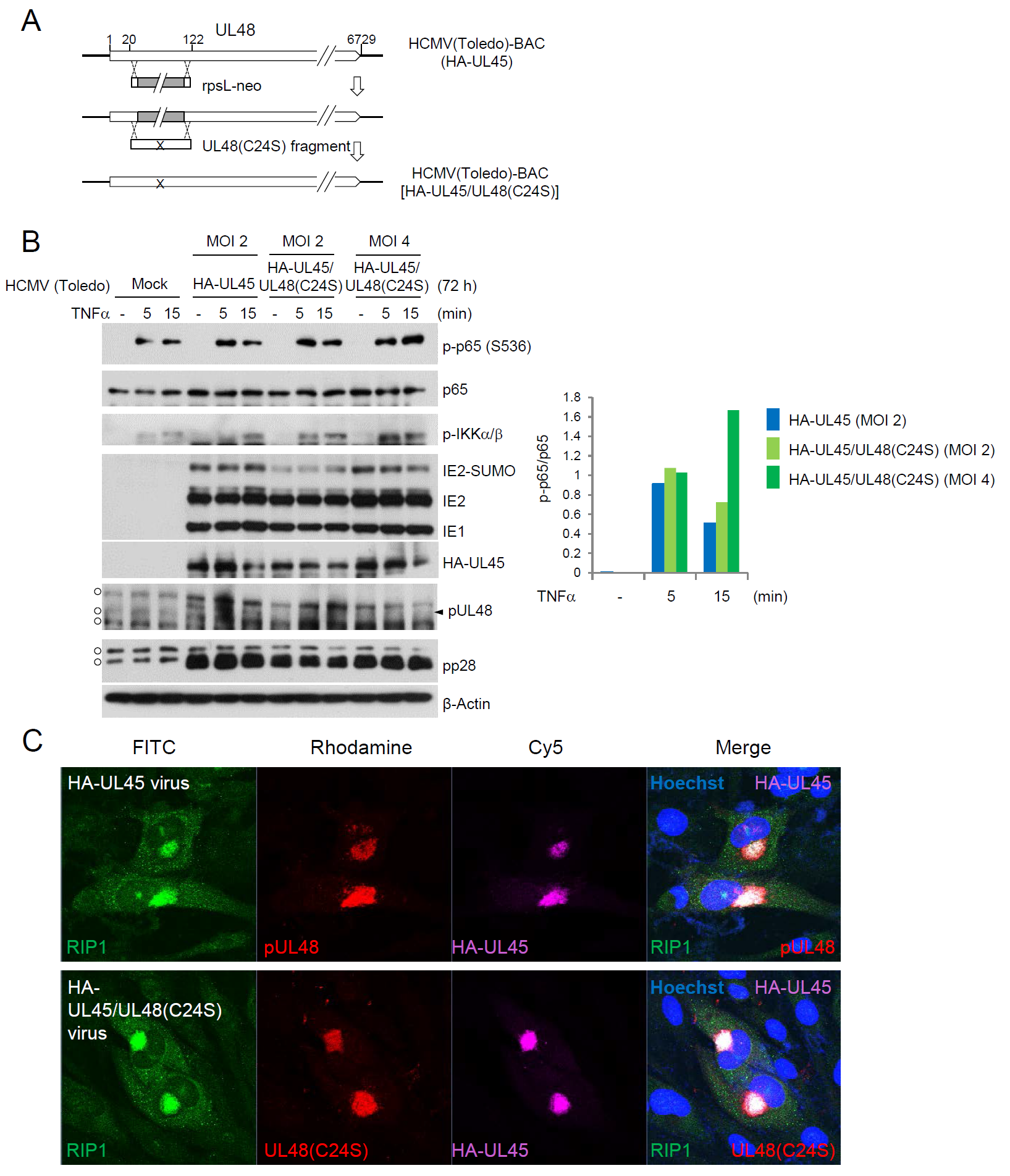

Supplement: S5 Fig — (A) The HCMV (Toledo) bacmid containing the UL48(C24S) gene was generated from the HA-UL45 bacmid using a counter-selection BAC modification kit (Gene Bridges) as in S2A Fig. First, the rpsL-neo cassette DNA was PCR-amplified using LMV2126/2127 primers containing homology arms and introduced into E. coli DH10B containing the HA-UL45 Toledo-BAC by electroporation to produce the rpsL-neo cassette-containing intermediate BAC constructs. Second, the UL48(C24S) fragments for replacing the rpsL-neo cassette were amplified by PCR using LMV2128/2129 primers and introduced into the rpsL-neo cassette-containing intermediates. The HA-UL45/UL48(C24S) Toledo-BAC clone was selected on LB plates containing streptomycin. LMV primers used for bacmid mutagenesis were as follows: LMV2126, 5’-GCTGCCACCAGGGCGACATCGCCCGCTTTGGAGCGCGAGCGGGCAATCAAGGCCTGGTGATGATGGCGGGATCG-3’; LMV2127, 5’- CTCGTTCCACCCAGGTGCAAGGCGTGTAGGAACATGATGCCGTTGCAGACTCAGAAGAACTCGTCAAGAAGGCG-3’; LMV2128, 5’- GCTGCCACCAGGGCGACATCGCCCG-3’; and LMV2129, 5’-CTCGTTCCACCCAGGTGCAAGGCGT-3’. (B) HF cells were mock-infected or infected with HA-UL45 virus at an MOI of 2 or with HA-UL45/UL48(C24S) virus at an MOI of 2 or 4 for 72 h. Cells were treated with TNFα (50 ng/ml) for 5 or 15 min. Total cell lysates were prepared and immunoblotting was performed with antibodies for p-p65(S536), p65, p-IKKα/β, anti-IE1/IE2, HA-UL45, UL48, pp28, or β-actin. Non-specific bands were denoted by open circles. The levels of p65 and phosphorylated p65 were quantitated by counting using ImageJ (NIH) and the changes of the ratio of phosphorylated p65 over p65 are shown as a graph. (C) HF cells were infected with HA-UL45 or HA-UL45/UL48(C24S) viruses for 96 h at an MOI of 1 and triple-label IFA was performed as in Fig 7C. (TIF) [file ppat.1006423.s005.tif]

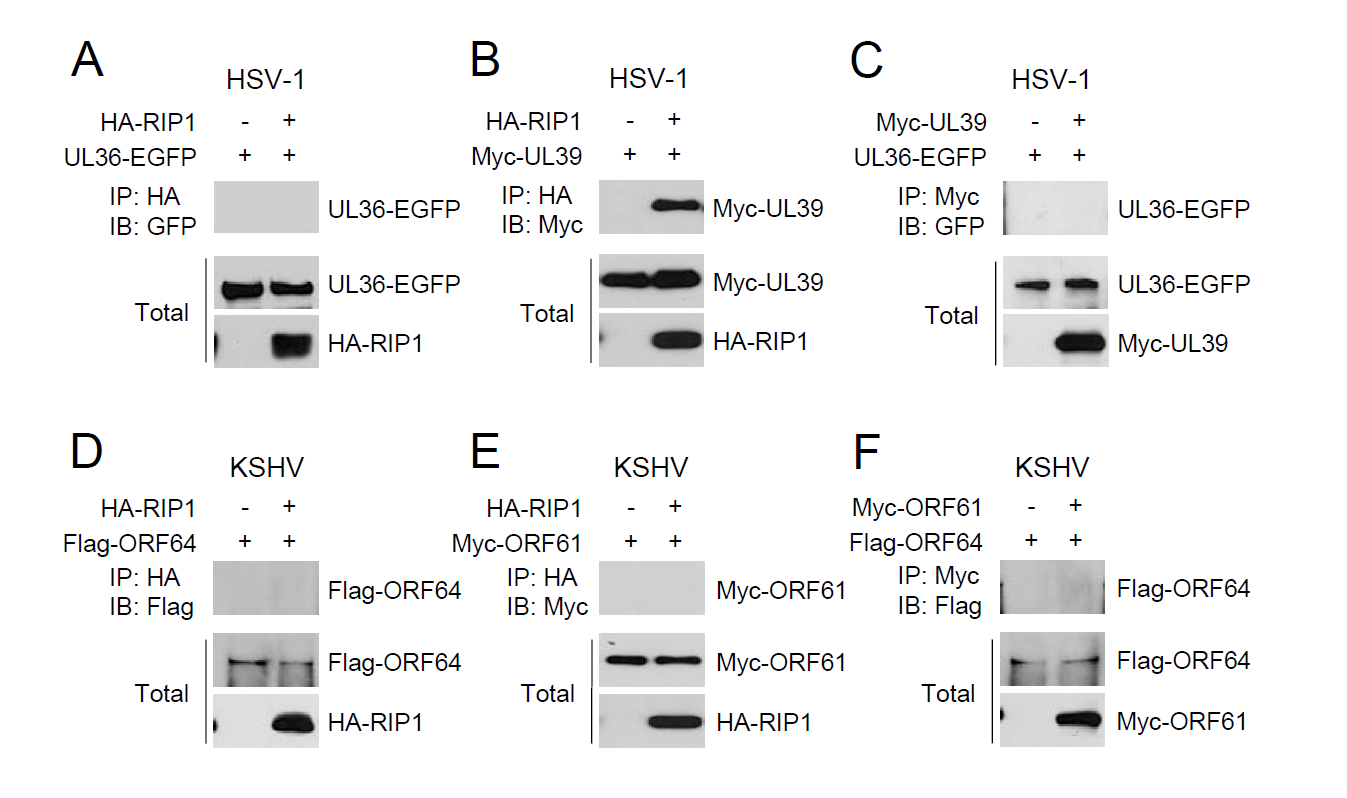

Supplement: S6 Fig — 293T cells were co-transfected with plasmid expressing HA-RIP1 and plasmids expressing HSV-1 proteins (UL36-EGFP and Myc-UL39) (A to C) or plasmids expressing KSHV proteins (Flag-ORF64, or Myc-ORF61 (D to F) as indicated. At 24 h after transfection, total cell lysates were immunoprecipitated with anti-HA or anti-Myc antibody and immunoblotting assays were performed as indicated. The protein levels in total cell lysates were also determined by immunoblotting. (TIF) [file ppat.1006423.s006.tif]

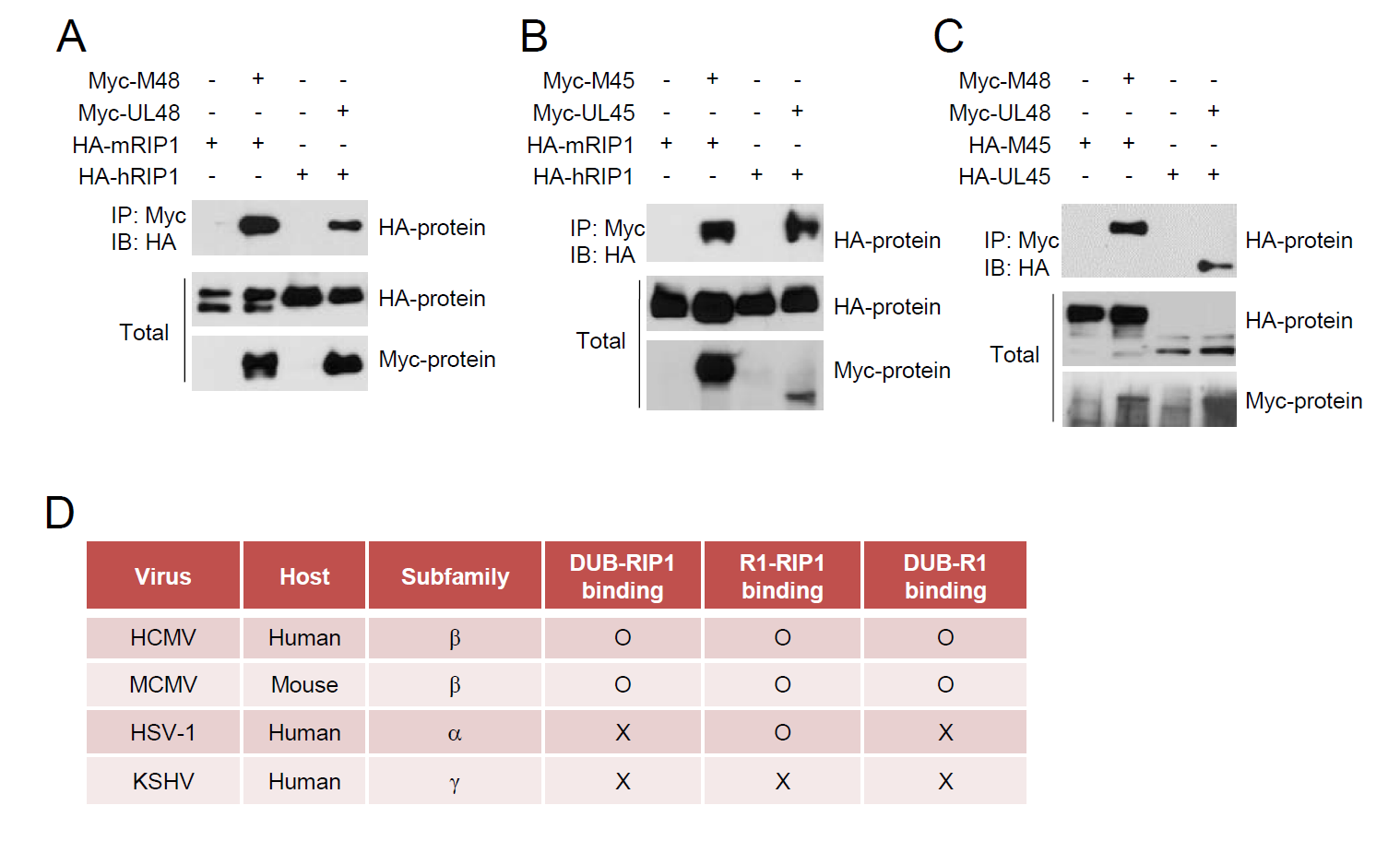

Supplement: S7 Fig — (A and B) 293T cells were co-transfected with plasmid expressing HA-RIP1 (mRIP1 or hRIP1) and plasmid expressing Myc-tagged viral DUB (M48 or UL48) (A) or plasmid expressing Myc-tagged viral R1 (M45 or UL45) (B), as indicated. At 24 h after transfection, total cell lysates were immunoprecipitated with anti-Myc antibody and immunoblotting assays were performed as indicated. The protein levels in total cell lysates were also determined by immunoblotting. (C) 293T cells were co-transfected with plasmids expressing Myc-tagged viral DUB (M48 or UL48) and HA-tagged viral R1 (M45 or UL45) as indicated. CoIP assays were performed as in (A). (D) Summary of the activity of viral DUB and R1 homolog to target RIP1 and interact with each other in different herpesviruses. (TIF) [file ppat.1006423.s007.tif]
